# Escape from X Inactivation Varies in Mouse Tissues

**DOI:** 10.1371/journal.pgen.1005079

**Published:** 2015-03-18

**Authors:** Joel B. Berletch, Wenxiu Ma, Fan Yang, Jay Shendure, William S. Noble, Christine M. Disteche, Xinxian Deng

**Affiliations:** 1 Department of Pathology, University of Washington, Seattle, Washington, United States of America; 2 Department of Genome Sciences, University of Washington, Seattle, Washington, United States of America; 3 Department of Medicine, University of Washington, Seattle, Washington, United States of America; University of Pennsylvania, UNITED STATES

## Abstract

X chromosome inactivation (XCI) silences most genes on one X chromosome in female mammals, but some genes escape XCI. To identify escape genes in vivo and to explore molecular mechanisms that regulate this process we analyzed the allele-specific expression and chromatin structure of X-linked genes in mouse tissues and cells with skewed XCI and distinguishable alleles based on single nucleotide polymorphisms. Using a binomial model to assess allelic expression, we demonstrate a continuum between complete silencing and expression from the inactive X (Xi). The validity of the RNA-seq approach was verified using RT-PCR with species-specific primers or Sanger sequencing. Both common escape genes and genes with significant differences in XCI status between tissues were identified. Such genes may be candidates for tissue-specific sex differences. Overall, few genes (3–7%) escape XCI in any of the mouse tissues examined, suggesting stringent silencing and escape controls. In contrast, an in vitro system represented by the embryonic-kidney-derived Patski cell line showed a higher density of escape genes (21%), representing both kidney-specific escape genes and cell-line specific escape genes. Allele-specific RNA polymerase II occupancy and DNase I hypersensitivity at the promoter of genes on the Xi correlated well with levels of escape, consistent with an open chromatin structure at escape genes. Allele-specific CTCF binding on the Xi clustered at escape genes and was denser in brain compared to the Patski cell line, possibly contributing to a more compartmentalized structure of the Xi and fewer escape genes in brain compared to the cell line where larger domains of escape were observed.

## Author Summary

X inactivation is a female-specific phenomenon that occurs during early development and results in the silencing of one X chromosome in female mammals. However, some genes escape inactivation and remain expressed from both X chromosomes. To date, the identity of escape genes and the molecular mechanisms of this process are still being explored. Here, we use a new binomial model combined with a mouse system with identifiable alleles and skewed X inactivation to identify and further define the chromatin landscape of escape genes in vivo. We find that some escape genes are common to multiple tissues while others are tissue-specific. We also show that expression levels of alleles on the inactive X correlate with factors associated with open chromatin such as RNA Polymerase II and DNase I hypersensitive sites. Additionally, escape genes co-localized with CTCF binding clusters on the Xi, suggesting a role for CTCF binding in delineating regions of escape and inactivation. Our findings represent the first comprehensive analysis of escape in vivo. Identification of tissue-specific escape genes could lead to a better understanding of the underlying causes of sex-linked disorders such as X-linked intellectual disability and Turner syndrome.

## Introduction

Dosage compensation in mammals is achieved by upregulation of the X chromosome in both sexes and random inactivation of one of the two X chromosomes in females [1]. Initially, the future inactive X (Xi) is coated by the long non-coding RNA *Xist* (X inactive specific transcript), a process essential for the onset of silencing [2]. Inactive chromatin marks such as tri-methylation of lysine 27 on histone H3 (H3K27me3) are put in place along with DNA methylation at CpG islands, macroH2A modification, and late replication, which represent late and possibly secondary events that lock in silencing of most genes in somatic cells. Despite efficient silencing some genes escape X chromosome inactivation (XCI) and thus remain bi-allelically expressed in females [3,4]. Surveys in cultured human/mouse hybrid cells and in cell lines from individuals with skewed XCI have shown that about 8–15% of human genes consistently escape XCI, 10–13% display variable levels of escape, and 10–20% vary between cell lines and individuals [5–7]. Escape from XCI results in significant sexual dimorphisms in levels of gene expression, and bi-allelic expression of at least some escape genes is important for a normal phenotype in human females. Indeed, the presence of a single X chromosome (45,X) results in Turner syndrome characterized by poor viability *in utero*, infertility, short stature, and an array of other abnormalities [8,9].

Genes that escape XCI usually lack both *Xist* RNA coating [10–12] and repressive histone modifications associated with silencing [13–18]. Furthermore, escape genes have specific DNA methylation signatures [19,20]. Whether other specific chromatin elements such as CTCF may be implicated is still unclear [21,22]. Little is known about the distribution of escape from XCI in different tissues in vivo and about the mechanisms that control tissue-specific differences.

XCI is usually random in somatic cells, thus allelic characteristics of X-linked genes can only be studied in cell populations with skewed XCI obtained by cell-cloning or flow-sorting, or by cell selection based on a specific mutation. To derive a cell line (Patski) with completely skewed XCI we previously used an *Hprt* mutation to select cells that contain an active X (Xa) from *Mus spretus* (abbreviated *spretus*) and an Xi from a *Mus musculus* laboratory strain (C57BL/6J abbreviated BL6) [23]. This in vitro system allowed us to determine allele-specific gene expression based on frequent SNPs (single nucleotide polymorphisms) between the mouse species (1/50–100bp), and to identify genes that escape XCI [18]. Mouse trophoblastic cells in which the paternal X is always inactivated offer an alternative way to identify genes that escape imprinted XCI in vitro [14]. However, Patski cells and trophoblastic cells may not represent the in vivo situation and do not address potential differences between tissues. A recent study examined mid-gestation placenta to address escape from imprinted XCI in vivo; but this system only addresses imprinted XCI in an extra-embryonic tissue, the mechanism of which differs from random XCI in the embryo proper [24]. Another recent study examined escape from XCI in mouse brain based on flow sorting cell populations with skewed XCI to >98% purity [25].

To determine the XCI status of genes in multiple tissues in vivo we developed a mouse model in which F1 animals have completely skewed XCI of the *spretus* X due to an *Xist* mutation on the BL6 X [26]. In the present study this model was exploited to compare the XCI status of genes between mouse tissues. We developed a new binomial model to estimate the probability of bi-allelic expression based on RNA-seq and SNPs, resulting in the identification of common as well as tissue-specific escape genes, which were verified by RT-PCR. Allele-specific profiles for two features of active genes, RNA polymerase II occupancy and DNase I hypersensitivity, demonstrate active chromatin signatures at escape genes. In addition, allele-specific profiles of CTCF occupancy were obtained to examine its distribution relative to escape genes.

## Results

### Pipeline to determine allele-specific gene expression

To assess allele-specific expression of X-linked genes in vivo, we mated BL6 females heterozygous for a deletion of the proximal A-repeat of *Xist* (*Xist* ^Δ/+^) [27] to *spretus* males. In the resulting F1 *Xist* ^Δ/+^ (thereafter called F1) female progeny the maternal BL6 X chromosome fails to inactivate, leading to completely skewed XCI in all tissues [26]. This was further verified based on allelic expression of a gene known to be subject to XCI (*Ubqln2*), as determined by Sanger sequencing of RT-PCR products (S1A Fig). Whole brains and spleens from two adult F1 females (biological replicates) and both ovaries pooled from one of these females were used for RNA-seq, followed by gene expression analyses using a new pipeline (see below) to better capture allele-specific reads based on high quality SNPs. For comparison and validation we also re-analyzed two independent RNA-seq datasets generated for the Patski cell line in which the XCI pattern is reversed, i.e. the BL6 X is inactive and the *spretus* X active [18].

To identify reads that map to each parental genome in F1 mice, a "pseudo-*spretus*" genome was assembled by substituting known SNPs between BL6 and *spretus* into the BL6 mm9 reference genome [26,28]. SNPs were obtained from the Sanger Institute (SNP database Nov/2011 version) and from in house analysis [18]. RNA-seq reads were aligned separately to the BL6 and to the pseudo-*spretus* genomes (see details in Material and Methods). We segregated all high-quality uniquely mapped reads (MAPQ ≥ 30) into three categories: (1) BL6-SNP reads containing only BL6-specific SNP(s); (2) *spretus*-SNP reads containing only *spretus*-specific SNP(s); (3) reads that do not contain valid SNPs. We refer to both BL6-SNP reads and *spretus*-SNP reads as "allele-specific reads". Examination of the distribution of allele-specific reads on genes known to be subject to XCI, for example *Ubqln2*, confirmed the absence of *spretus* reads due to XCI skewing in female F1 tissues (S1B Fig).

We calculated diploid gene expression based on all mapped reads using cufflinks/v2.0.2 [29] (http://cufflinks.cbcb.umd.edu/) to determine RPKM (reads per kb of exon length per million mapped reads). Next, we defined SNP-based haploid gene expression from alleles on the Xi or the Xa (Xi-SRPM or Xa-SRPM) to be allele-specific SNP-containing exonic reads per 10 million uniquely mapped reads.

### Modeling the XCI status of genes

Here, we propose and validate a new binomial model to identify escape genes and estimate the statistical confidence of escape probability.

For each gene *i* on chromosome X, let the number of allele-specific RNA-seq reads mapped to the inactive/active chromosomes be *n*_*io*_ and *n*_*i*1_, respectively, and let. *n*_*i*_ = *n*_*io +*_ *n*_*i*1_ We model *n*_*io*_ by a binomial distribution

$$n_{i0}\sim \text{Binomial}\left(n_{i},p_{i}\right)$$

where *p*_*i*_ indicates the expected proportion of reads from the Xi. The estimate of the binomial proportion is

$$\hat{p_{i}}=n_{i0}/n_{i}$$

Let *z*α/2 be the 100(1 - *α*/2)^*th*^ percentile of *N*(0,1). The confidence interval of each $\hat{p_{i}}$ is,

$$\hat{p_{i}}\pm z_{\alpha /2}\sqrt{\hat{p_{i}}\left(1-\hat{p_{i}}\right)/n_{i}}$$

To incorporate the mapping biases toward the BL6 genome over the pseudo-*spretus* genome into the above model, we define the mapping bias ratio *r*_*m*_ for each RNA-seq experiment to be

$$r_{m}=N_{A0}/N_{A1}$$

where *N*_*A0*_ and *N*_*A1*_ are the number of allele-specific autosomal reads in the "inactive X containing" genome and the "active X containing" genome, respectively. Considering the mapping biases the corrected estimate of *p*_*i*_ is:

$$\bar{p_{i}}=n_{i0}/\left(n_{i0}+r_{m}n_{i1}\right)=\hat{p_{i}}/\left(\hat{p_{i}}+r_{m}\left(1-\hat{p_{i}}\right)\right)$$

The upper and lower confidence limits are corrected accordingly.

**Fig 1:**
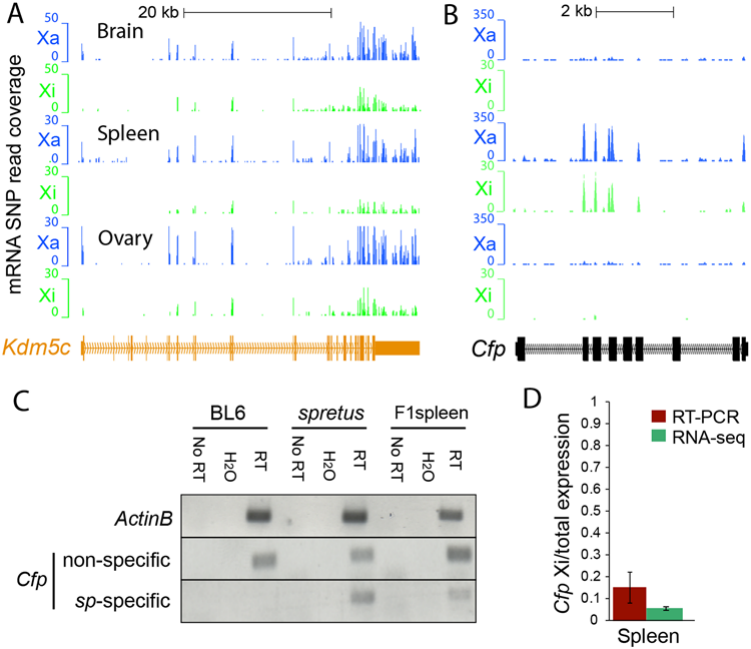
Evaluation of escape from XCI in mouse tissues. (A) Example of mRNA SNP read distribution profiles on the Xi and Xa for *Kdm5c*, an escape gene common to all mouse tissues tested (brain, spleen and ovary). SNP reads specific to the Xa (blue) and Xi (green) are visualized in the UCSC genome browser. RNA-seq read quantification was done by normalizing reads from the Xi to total reads (Xi + Xa) in two biological replicates. (B) Example of mRNA SNP read distribution profiles on the Xi and Xa for *Cfp*, a gene that escapes XCI only in spleen. SNP reads specific to the Xa (blue) and Xi (green) are visualized in the UCSC genome browser. (C, D) Validation of escape from XCI for *Cfp*. (C) Gel electrophoresis of RT-PCR products using non-species-specific primers and *spretus*-specific primers (sp) (S10 Table) in BL6, *spretus*, and F1 brain in which the Xi is from *spretus*. *ActinB* was used as a control. Control reactions include "No RT" (no reverse transcriptase) and H_2_O (instead of primers). (D) Graph comparing RT-PCR *Cfp* gel band quantification measured by ImageJ with SNP read quantification measured by RNA-seq. Xi product abundance measured by RT-PCR using *spretus*-specific primers in F1 spleen was normalized to total RT-PCR product abundance measured by non-species specific primers.

**Fig 2:**
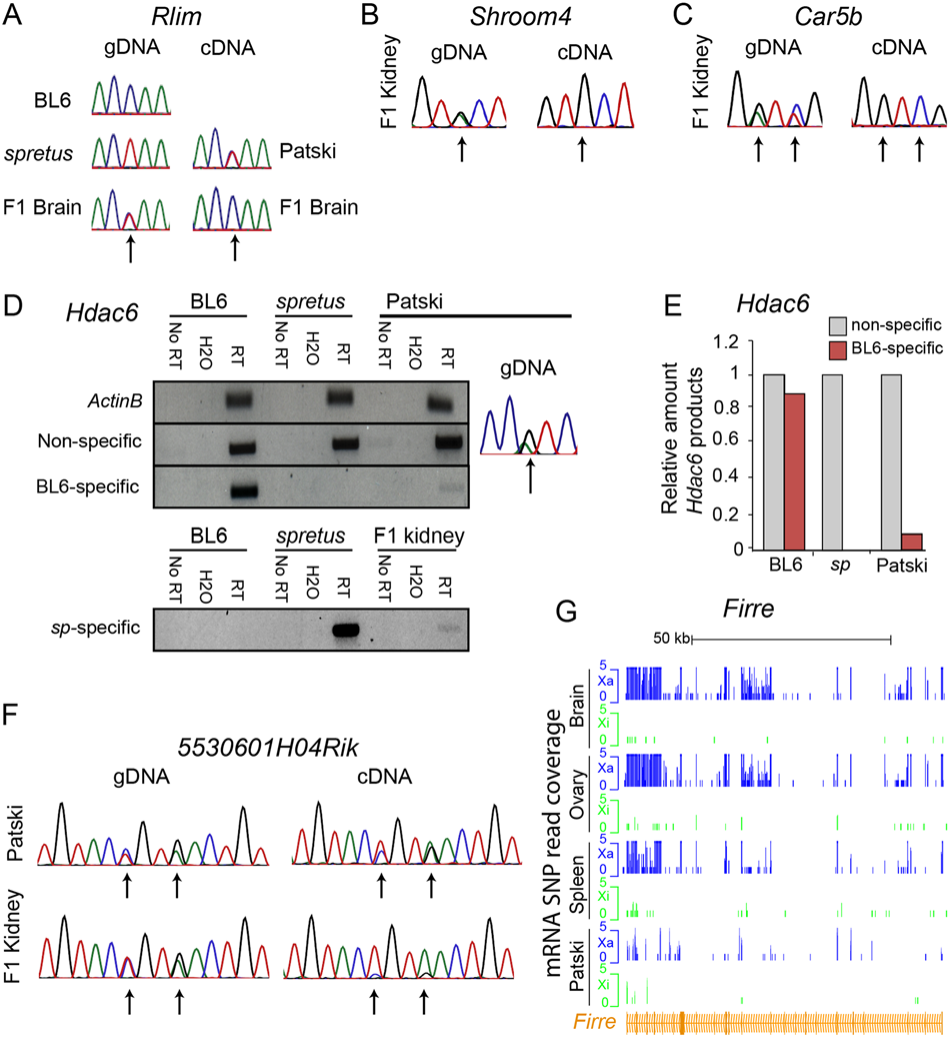
Validation of *Rlim*, *Shroom4*, *Car5b*, *Hdac6*, *5530601H04Rik* expression profiles and *Firre* mRNA profiles. (A) Sanger sequencing tracings of *Rlim* cDNA confirm bi-allelic expression in Patski cells but not brain, while gDNA sequence tracings show SNP heterozygosity (C in BL6 and T in *spretus*). Arrows indicate SNP positions. (B, C) Sanger sequencing tracings of *Shroom4* (B) and *Carb5* (C) cDNA confirm that these genes are subject to XCI in F1 kidney while they were shown to escape XCI in Patski cells [18]. gDNA sequence tracings show SNP heterozygosity (*Shroom4*—G in BL6 and A in *spretus*; *Car5b*—G and C in BL6; A and T in *spretus*). Arrows indicate SNP positions. (D, E) Validation of escape from XCI for *Hdac6*. (D) Gel electrophoresis of RT-PCR products using non-species-specific primers, *spretus*-specific primers, and BL6-specific primers (S10 Table) in BL6, *spretus*, Patski cells and F1 kidney. *ActinB* was used as a control. Control reactions include "No RT" (no reverse transcriptase) and H_2_O (instead of primers). Sanger sequencing tracing confirms heterozygosity (A in BL6 and G in *spretus*) in the left primer (S10 Table). (E) Xi expression of *Hdac6* was determined to be 9% of total expression in Patski cells by gel band quantification measured by ImageJ. (F) Sanger sequencing tracings of *5530601H04Rik* cDNA confirms that the lncRNA escapes XCI in kidney and Patski cells, while gDNA sequence tracings show heterozygosity (T and A in BL6; A and G in *spretus*). Arrows indicate SNP positions. (G) mRNA SNP read distribution profiles on the Xi and Xa for *Firre*, a lncRNA that escapes XCI in mouse tissues and Patski cells. Note that *Firre* is classified as a variable escape gene in brain (S1 Dataset). Xa SNP reads are in blue and Xi SNP reads in green.

**Fig 3:**
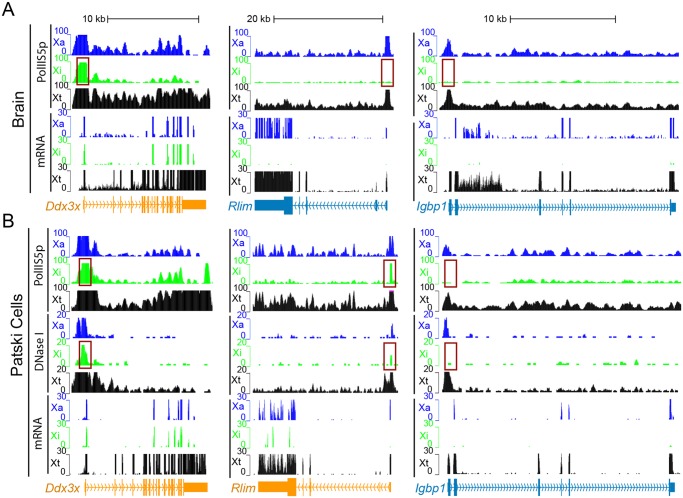
Enrichment in PolII-S5p and DNase I hypersensitivity on the Xi allele at escape genes. (A, B) Examples of allele-specific PolII-S5p occupancy profiles and expression (mRNA) profiles at *Ddx3x*, *Rlim* and *Igbp1* in two systems: brain (A) and Patski cells (B). *Ddx3x* escapes XCI in both systems, *Rlim* escapes XCI in Patski cells only, and *Igbp1* is subject to XCI in both systems. PolII-S5p is enriched at the promoter regions (highlighted by a red box) of escape genes on both the Xa and the Xi, whereas enrichment is limited to the Xa for genes subject to XCI. DNase I hypersensitivity tested in Patski cells only is also increased at the promoter regions (highlighted by a red box) of escape genes on both the Xa and Xi, but is limited to the Xa for genes subject to XCI. Genes that escape XCI are labeled orange and genes subject to XCI blue. Color-coded profiles are shown for the Xa (blue) and Xi (green) SNP reads, and for the total reads (Xt, black). See additional examples in S2 Fig.

For each RNA-seq experiment, we called a gene "*escape*" if (1) the 99% lower confidence limit (*α* = 0.01) of the escape probability was greater than zero, indicating significant contribution from the Xi, (2) the diploid gene expression measured by RPKM was ≥1, indicating that the gene was expressed, and (3) the Xi-SRPM was ≥2, representing sufficient reads from the Xi. Note that we only considered exonic reads, except for the lncRNA *Firre* (see below). Biological replicates of RNA-seq experiments were analyzed separately. Examples of mRNA SNP-read coverage on the Xa and Xi visualized in the UCSC browser are shown for a number of genes we determined to either escape or be subject to XCI (Figs. 1–3, S1, S2 and S4). There was a good concordance between reads at different SNPs within a given gene. Levels of escape from XCI determined by RNA-seq analysis correlated with measurements done by RT-PCR using species-specific primers, although the percent of expression from the Xi measured by RT-PCR was usually higher than that determined by RNA-seq. For example, *Cfp* and *Plp1* had 5% and 0.3% SNP reads on the Xi by RNA-seq and 15% and 1.5% Xi (versus Xa) expression measured by RT-PCR with species-specific primers, respectively (Figs. 1C and 1D, S1C and S1D).

### Classification of mouse escape genes

We determined that 3–5% of X-linked Refseq genes satisfy our criteria (see above) as escape genes in both biological replicates of F1 brain and spleen, respectively (S1–S3 Table). In addition, 3% of X-linked genes variably escaped XCI, i.e., escaped in only one biological replicate. In F1 ovary 7% of X-linked genes escaped XCI (S1 and S4 Table), possibly due to the analysis of a single replicate for this tissue (representing two pooled ovaries). Escape genes were distributed all along the mouse X chromosome and rarely clustered, and distance from the XIC (X inactivation center) did not appear to influence levels of expression from the Xi. We classified escape genes into two groups based on their XCI status, excluding genes with a different status in biological replicates: group 1 includes 12 genes that escape XCI in at least two of the three tissues analyzed and group 2, 26 genes that escape XCI in a tissue-specific manner (Table 1). When considering potential sex bias in gene expression we found that based on published data [30], a majority (27 of 38 or 71%) of group1 and 2 escape genes showed a female bias and thus may play roles in sex differences (S5 Table).

**Table 1:** Escape genes grouped as common or variable between mouse tissues.

	Gene	Brain SRPM (Xi/Xa)	Brain RPKM	Spleen SRPM (Xi/Xa)	Spleen RPKM	Ovary SRPM (Xi/Xa)	Ovary RPKM	Escape in Human
**Group 1**	*Ddx3x*	56/178	85	76/215	120	66/216	120	9/9
	*Kdm6a*	6/18	10	25/39	28	13/28	17	9/9
	*Eif2s3x*	130/356	65	267/588	109	133/379	77	9/9
	*Xist*	1755/4	50	2832/7	89	1849/7	60	9/9
	*5530601H04Rik*	8/28	3	7/37	5	10/24	3	-
	*Pbdc1*	2/20	6	7/30	17	10/26	11	0/9
	*Kdm5c*	19/64	10	43/120	23	32/91	18	9/9
	*Cybb*	0/3	0	14/607	55	2/31	3	-
	*Utp14a*	2/31	9	5/119	35	4/47	15	3/9
	*Ftx*	9/115	12	4/87	10	4/34	4	-
	*Firre*	2/208	11*	6/74	4*	5/495	29*	-
	*Slc16a2*	2/39	7	2/11	2	3/38	8	0/5
**Group 2**	*Plp1*	13/4561	901	0/7	2	0/25	6	1/9
	*Gpm6b*	35/2557	334	0/81	12	0/101	16	9/9
	*Syp*	10/1662	358	0/7	2	0/8	2	2/9
	*Gdi1*	4/439	144	1/141	53	1/135	47	-
	*Gprasp1*	3/1775	136	0/86	8	2/363	32	-
	*Tmem47*	5/320	45	0/18	3	1/121	20	0/9
	*Cfp*	0/12	6	12/210	131	1/18	11	0/9
	*Bgn*	1/14	5	5/332	91	1/218	69	0/6
	*Vsig4*	0/0	0	2/3	2	0/1	0	-
	*5730416F02Rik*	0/0	0	6/3	6	2/1	3	-
	*5430427O19Rik*	0/1	0	4/79	9	1/5	0	-
	*Lamp2*	0/168	43	0/318	79	3/709	183	0/9
	*AU015836*	0/0	0	0/0	0	4/6	3	-
	*Kif4*	0/3	0	0/182	21	2/34	5	0/4
	*Rlim*	1/187	13	1/282	23	2/209	19	0/9
	*Sh3bgrl*	0/290	46	1/402	71	3/847	148	8/9
	*Fam199x*	0/65	4	0/35	2	2/57	4	0/9
	*Tmem164*	0/87	7	2/154	14	3/229	21	-
	*Alg13*	0/15	4	0/17	5	2/29	8	5/9
	*Tmem29*	1/24	16	1/16	13	2/18	17	0/5
	*Pdha1*	2/575	106	1/264	57	2/725	157	0/6
	*AU022751*	0/0	0	0/0	0	11/11	6	-
	*Rnf128*	0/33	8	0/3	1	9/262	79	-
	*Car5b*	0/9	1	0/6	1	10/281	42	9/9
	*Bmp15*	0/0	0	0/0	0	25/24	8	-
	*Flna*	0/76	10	1/616	82	5/859	120	0/9

Gene names are listed to reflect their classification into group 1 (common in at least two tissues) or group 2 (escape in only one tissue). Tissue-specific escape genes escape XCI in 1 of 3 tissues but not in any replicate of other tissues. For brain and spleen average SRPM-Xi/Xa values between replicates are shown. Ovary SRPM-Xi/Xa values represent one sample. Gene expression is shown as RPKM. Whether human homologs to the mouse genes escape XCI is shown in the last column, which indicates the proportion of mouse x human hybrid cell lines that had expression of the gene from the human Xi [6]. A dash indicates the gene was not assayed for escape in human in that study. *Firre* RPKM (*) was based on reads from exons and introns (see also S2–4 Table and Methods).

Of the 12 common escape genes classified as belonging to group 1, only the lncRNA *5530601H04Rik* was not included in our original survey in Patski cells [18], although our current re-analysis now includes it (see below). *Mid1*, a gene that straddles the boundary of the pseudoautosomal region (PAR) in *M*. *musculus* and escapes XCI in Patski cells [18] was not classified as an escape gene in our current analysis of mouse tissues with a *spretus* Xi, indicating that *Mid1* is subject to XCI in *M*. *spretus* where it is located outside the PAR [31]. This was confirmed in F1 brain by Sanger sequencing of RT-PCR products (S1E Fig).

Within group 2 a total of 6, 5, and 15 genes escaped XCI selectively in F1 brain, spleen, and ovary, respectively. The findings of escape in a single tissue often reflected the unique expression pattern of these genes. Functional analysis showed that group 1 common escape genes often have functions relevant to many tissues, whereas group 2 genes have tissue-specific functions (S5 Table). In fact, all 6 genes that escape XCI in brain have brain-related functions, for example, *Gpm6b* and *Plp1* play a role in myelination [32]. RT-PCR validation using species-specific primers confirmed *Plp1* expression from the Xi in brain (S1C and S1D Fig). In addition, *Gdi1* and *Syp* have been implicated in X-linked intellectual disability in humans [33,34], and *Gprasp1* mutations are associated with striatum-dependent behavior inhibition [35]. In spleen a total of 17 escape genes were identified, including 5 spleen-specific escape genes, three of which, *Cfp*, *Vsig4*, and *Bgn*, implicated in immune functions [36–39] (Tables 1, S3 and S5). *Cfp* escaped from XCI in spleen but not in brain or ovary, as validated by RT-PCR using species-specific primers (Table 1 and Fig. 1B–1D). *Vsig4* expression from the Xi in spleen was verified by Sanger sequencing of RT-PCR products, but in liver where *Vsig4* is also highly expressed this gene was subject to XCI, suggesting that escape in spleen was independent of tissue-specific expression (S1F and S1G Fig). In ovary a total of 33 escape genes were identified, including 15 ovary-specific escape genes (Tables 1 and S4). Note that six additional genes were not included in Table 1 since they escaped in only one biological replicate of ovary (*Idh3g*, *Mmgt1*, *Usp9x*, *Uba1*, *Huwe1*, *1810030O07Rik*). Among the ovary-specific escape genes, *AU022751* and *Bmp15* had nearly equal expression from the Xa and Xi, as confirmed by Sanger sequencing of RT-PCR products (Table 1, S1H and S1I Fig). Since both *AU022751* and *Bmp15* are mostly expressed in oocytes where the Xi is reactivated [40] the finding of bi-allelic expression in ovary was not surprising. X reactivation could account for the larger number of escape genes in this tissue, even for genes that are not exclusively expressed in oocytes (S4 Table).

Using our new pipeline to re-analyze two independent RNA-seq datasets for Patski cells, one previously generated in our lab [18] and the other generated on an AB Solid platform deposited for ENCODE [41], we identified 66 escape genes (S1 and S6 Table). Fifty-two of these genes did not escape in any of the three F1 tissues (S2–S4 and S6 Table). For example, *Rlim* that escaped XCI in Patski cells was subject to XCI in brain (Fig. 2A). Next we examined gene expression in F1 kidney since Patski cells were derived from 18.5dpc embryonic kidney [23]. We found that two genes that escaped XCI in Patski cells, *Shroom4* and *Car5b* (as seen here and in [18]), failed to express from the Xi in kidney, indicating that these genes escaped XCI only in the cell line (Figs. 2B, 2C and S4). In contrast, RT-PCR analyses using species-specific primers for *Hdac6* showed that this gene escaped XCI both in Patski cells and kidney, suggesting that this gene is a tissue-specific escape gene since it does not escape XCI in other tissues (Fig. 2D, 2E and Table 1). The lncRNA *5530601H04Rik* was confirmed to escape XCI in both Patski cells and kidney using Sanger sequencing of RT-PCR products, indicating that this is a common escape gene in all tissues examined (Fig. 2F and Table 1).

The larger number of genes classified as "escape" in the new analysis of Patski cells compared to our previously published data [18] was mainly attributed to our new binomial model and not to differences in SNP number (see details in Methods). While our previous study used a ratio of Xi/Xa SNP reads greater than 0.1 (10% Xi expression) in the entire gene body to call a gene escape, our current binomial model to compare Xi-SNP reads to total SNP reads in exons more accurately assesses Xi expression, as shown by our verifications using RT-PCR with species-specific primers or Sanger sequencing (see above). Two of three genes previously classified as "escape" [18], *Bgn* and *BC022960*, were not included in our current list because their expression was below the 1RPKM threshold. The lncRNA *Firre* (*6720401G13Rik*) was initially excluded in our current analysis based on exonic SNPs because it failed to pass the ≥2 Xi-SRPM cutoff in tissues and in one biological replicate of Patski cells. However, when intronic SNPs were considered, similar to our previous survey [18] *Firre* was re-classified as an escape gene in F1 tissues and in both replicates of Patski cells (Fig. 2G, Tables 1 and S2–S4 and S6). This is also supported by another study [42], and by our findings of enrichment in RNA polymerase II phosphorylated at serine 5 (PolII-S5p) within the gene body of *Firre* on the Xi, suggesting alternative transcript start sites on the Xi (Yang et al., manuscript in review).

### Enrichment in RNA polymerase II and DNAse I hypersensitivity at escape genes

**Fig 4:**
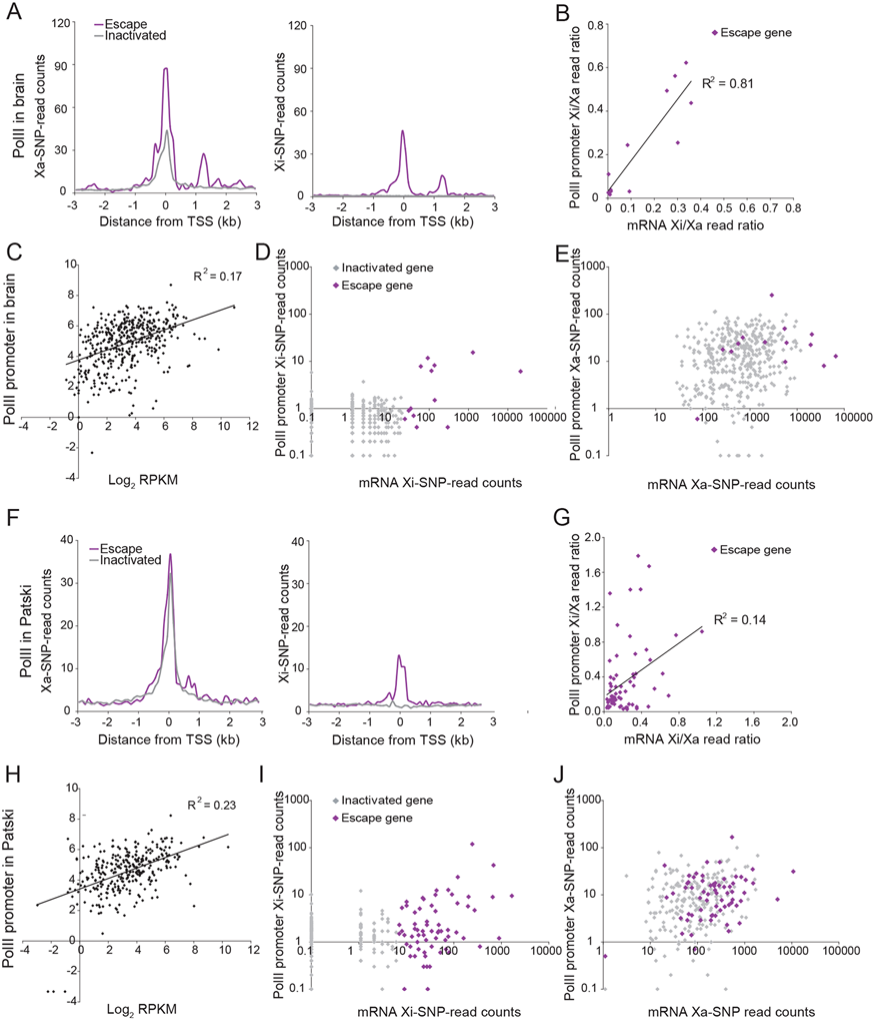
PolII-S5p enrichment at promoters of X-linked genes on the Xi correlates with expression and escape from XCI. (A) Metagene analyses in brain show average Xa- (left) and Xi- (right) SNP read counts in 100bp windows 3kb upstream and downstream of the TSS for escape genes (purple; 14 genes with ≥2 Xi-SRPM in both replicates for brain) and for genes subject to XCI (gray; 403 genes with <2 Xi-SRPM in both replicates for brain). (B) The ratios of PolII-S5p enrichment at the promoter (SNP reads within ±500bp of the TSS) of escape genes (purple) on the Xi versus the Xa are strongly correlated to the ratios of expression from the Xi versus the Xa measured by RNA-seq in brain. *Xist* is excluded in this analysis. (C) Scatter plot of PolII-S5p promoter enrichment (log_2_ of reads within ±500bp of the TSS) against expression levels of all X-linked genes (log_2_ RPKM) shows a positive correlation in brain. (D) Scatter plot of Xi-specific PolII-S5p promoter enrichment (SNP reads within ±500bp of the TSS) against expression (Xi SNP reads) for escape genes (purple) and genes subject to XCI (gray) in brain. Promoter PolII-S5p enrichment correlates with expression from the Xi. (E) Same analysis as for D but for Xa-specific PolII-S5p promoter enrichment in brain. Escape genes generally overlap with genes subject to XCI, but are often highly expressed and enriched in PolII-S5p. (F-J). Same analyses as A-E for Patski cells.

The distribution of PolII-S5p was determined by ChIP-seq in one female F1 brain and in Patski cells in conjunction with re-analyses of two DNase I hypersensitivity datasets for Patski cells [41] to extract allele-specific profiles for comparison with the XCI status of genes. There was good agreement between bi-allelic promoter enrichment in PolII-S5p and escape from XCI, as shown for common escape genes *Ddx3x*, *Kdm6a* and *5530601H04Rik* (Figs. 3, S2). In contrast, genes subject to XCI in both brain and Patski cells such as *Igbp1* lacked these features on the Xi (Fig. 3). For genes that differed in terms of their XCI status in a tissue-specific manner, corresponding differences in PolII-S5p levels were noted between tissues. For example, PolII-S5p was bound only to the Xa allele of *Rlim* in brain where this gene is subject to XCI, while bi-allelic PolII-S5p enrichment was observed in Patski cells where the gene escapes XCI (Fig. 3). Similar results were seen at other differential escape genes, such as *Shroom4* (S2 Fig). Consistently, metagene analyses showed high PolII-S5p occupancy at the promoter regions of both alleles of escape genes but not of inactivated genes on the Xi in brain and Patski cells (Fig. 4A and 4F). For escape genes, there was a strong correlation between the ratio of PolII-S5p enrichment on the Xi versus the Xa with allele-specific expression levels (Fig. 4B and 4G). When examining all assessable X-linked genes, the level of PolII-S5p occupancy at the promoter was positively correlated with expression as expected (Fig. 4C and 4H). Allele-specific scatter plots showed a positive correlation between PolII-S5p enrichment at the promoter for Xi-alleles of escape genes, while no such correlation was observed for Xa-alleles (Fig. 4D, 4E, 4I and 4J). Interestingly, escape genes tend to have higher expression that inactivated genes (Fig. 4E and 4J). We did find four genes that were not classified as escape genes in our RNA-seq analyses, yet showed PolII-S5p occupancy on the Xi with an average read counts >5 at their promoter (0.5kb ± TSS) in brain (*Snx12*) and Patski cells (*Phf6*, *Trappc2* and *Usp9x*) (S2 and S3 Dataset). PolII-S5p Xi occupancy at *Trappc2* could be due to overlap of its promoter region with that of *Ofd1*, an escape gene in Patski cells (S6 Table). Note that *TRAPPC2* and *USP9X* have been reported to escape XCI in human [6].

**Fig 5:**
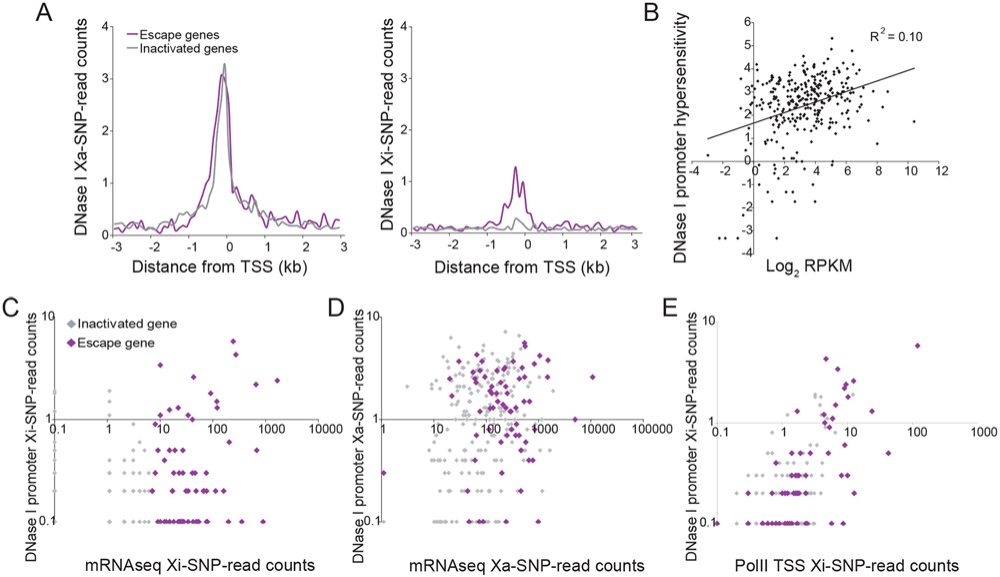
DNase I hypersensitivity at the promoters of X-linked genes correlates with expression and escape from XCI. (A) Metagene analyses of DNase I hypersensitivity (DHS) in Patski cells show average Xa- (left) and Xi- (right) SNP read counts in 100bp windows 3kb upstream and downstream of the TSS for escape genes (purple; 43 genes in both replicates for Patski cells) and for genes subject to XCI (gray; 203 genes with <2 Xi-SRPM in both replicates for Patski cells). (B) Scatter plot shows a positive correlation between DHS at the promoter (log_2_ of all reads within a region ±500bp from the TSS) and expression (log_2_ RPKM) for all X-linked genes in Patski cells. (C) Scatter plot of Xi-specific DHS at the promoter (reads within a region ±500bp from the TSS) against expression (Xi SNP-reads) for escape genes (purple) and genes subject to XCI (gray) shows a correlation between DHS and level of escape from XCI in Patski cells. (D) Same analysis as in C but for Xa-specific DHS at the promoter region. Escape genes generally overlap with genes subject to XCI although escape genes tend to have high expression and high DHS. (E). Scatter plot shows a good correlation between DHS and enrichment in PolII-S5p at the promoter of genes on the Xi. DHS and PolII-S5p are shown as reads within a region ±500bp from the TSS for escape genes (purple) and genes subject to XCI (gray) in Patski cells.

Analyses of promoter DNase I hypersensitivity in Patski cells showed similar correlations with X-linked gene expression as well as with Xi expression (Figs. 3, 5 and S2). In addition, there was a good correlation between DNase I hypersensitivity and enrichment in PolII-S5p at the promoter region of genes on the Xi (Fig. 5E). Thus, DNase I hypersensivity did not appear to be an "all or none" feature but rather was correlated to Xi expression level.

### Allelic CTCF binding analysis

While genes that escape XCI in F1 brain were few and almost all isolated those that escape XCI in Patski cells were more numerous and tended to cluster. When considering all escape genes in either replicate of Patski cells we identified 13 clusters with a high density of escape genes compared to the number of genes subject to XCI, including a 440kb cluster containing 13 escape genes (S6 Table and S1 Dataset). Furthermore, 22/66 escape genes in Patski cells were found within 12 (dark gray-shaded areas) of the 16 long-range *cis*-regions (i.e. 4C domains) previously shown to contain escape genes and to physically interact with *Cdk16* and/or *Kdm5c* in neural progenitor cells (S6 Table) [43]. To determine whether CTCF binding might contribute to differences in the number and distribution of escape genes between F1 brain and Patski cells allele-specific profiles of CTCF binding were generated by ChIP-seq. Discrimination between alleles using SNPs was verified by examining allele-specific CTCF binding at two imprinted autosomal regions known to differentially bind CTCF at the DMR (differentially methylated region). As previously demonstrated [44], *H19* showed maternal CTCF binding, while *Peg13* was bound by CTCF only on the paternal allele in both brain and Patski cells (S3A Fig).

For allele-specific peak calling all mapped reads were first used to identify enriched peak regions in the diploid genome. Two independent peak calling programs were applied, CisGenome (FDR cutoff 10^-5^) [45,46] and MACS/v1.4 (p-value cutoff 10^-5^) [47]. We defined significantly enriched peak regions as those identified by both peak callers. Next, we selected allele-specific ChIP-seq peaks using a binomial test. For each diploid ChIP-seq peak region, we assumed that the numbers of BL6-SNP reads (*n*_*i*_,_*bl*_) and *spretus*-SNP reads (*n*_*i*_,_*sp*_) within the peak follow a binomial distribution, i.e.,

$$n_{i,bl}\sim Binomial\left(n_{i},p_{i}\right)$$

where *n*_*i*_ = *n*_*i*_,_*bl*_ + *n*_*i*_,_*sp*_ is the sum of BL6-SNP reads and *spretus*-SNP reads in peak region *i*, and *p*_*i*_ is the binomial parameter. Since the X chromosome behaves differently from autosomes due to skewed XCI in our systems, we estimated the X chromosome allelic background using all SNP reads in the identified diploid peak regions on the X only. That is, for peaks on the X chromosome,

$$p_{i}=p_{X}=\frac{N_{X,bl}}{N_{X,bl}+N_{X,sp}}$$

in which *N*_*x*_,_*bl*_ and *N*_*x*_,_*sp*_ are the total number of BL6-SNP and *spretus*-SNP reads in X peaks, respectively. Finally, BL6-preferred ChIP-seq peaks were defined as those that contain significantly more BL6-SNP reads (upper-tail binomial test, p-value <0.05), while *spretus*-preferred ChIP-seq peaks were identified using the lower-tail binomial test (p-value <0.05), and both-preferred ChIP-seq peaks were those peaks that were not significant in the two above tests (p-value ≥0.25). In addition, we required the allele-assessable peaks have a minimal SNP read coverage of one allele-specific read (BL6-SNP and *spretus*-SNP reads) per 10 million mapped reads. Of the allele-assessable CTCF-binding peaks on the X chromosome in brain and Patski cells (1639/2263 and 374/532, respectively) we identified 212 (13%) Xi- and 366 (22%) both-preferred, and 86 (23%) Xi- and 161 (43%) both-preferred, respectively (S7 Table). The much larger number of CTCF peaks on the Xi in brain suggests a different structure of the Xi. In fact, only 62 of the Xi-binding CTCF peaks were common between brain and Patski cells (S7 Table).

**Fig 6:**
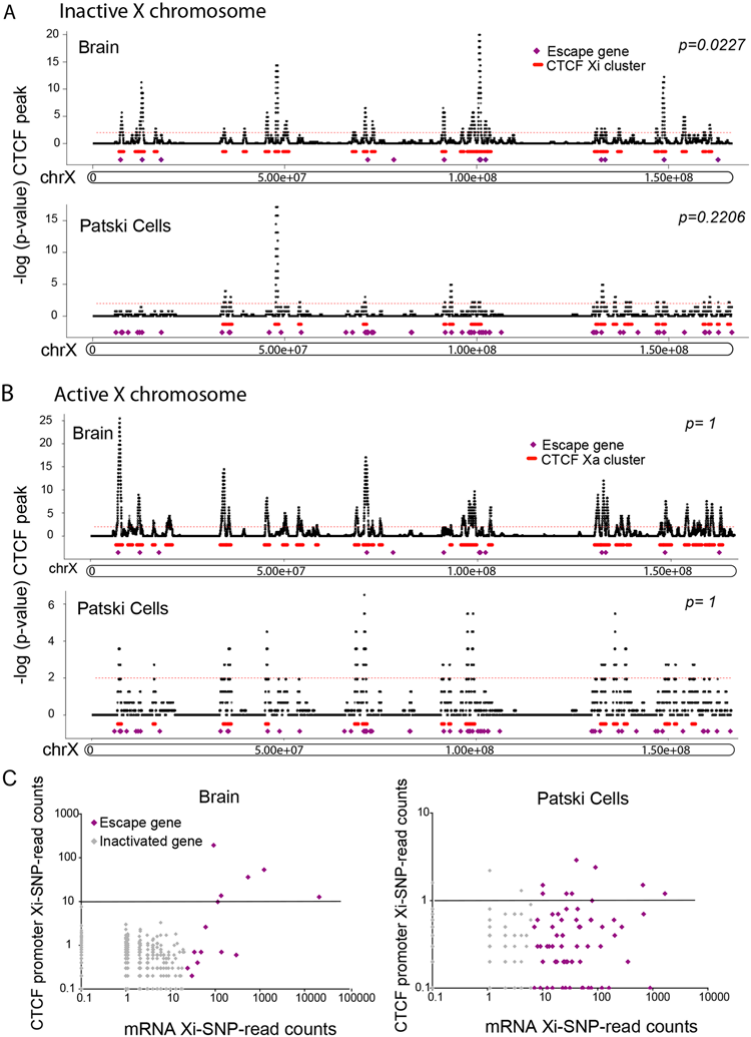
Xi-associated but not Xa-associated CTCF peak clusters co-localize with escape regions. (A) Significant CTCF Xi-binding clusters were mapped along the Xi in brain and Patski cells. Xi- and both-preferred peaks were determined by a binomial model and used for density analysis. Red bars represent merger of clusters of CTCF Xi-binding peaks, while purple dots represent escape genes. Significant Xi-binding CTCF binding clusters tend to co-localize with chromatin containing escape genes. Little change was seen after removal of promoter-associated CTCF binding (S3B Fig). Horizontal axis represents the Xi in Mb. The vertical axis is the negative log of the calculated binomial p-value (-log (p-value)). The thin red dashed line represents a 0.01 p-value cutoff. (B) Similar analysis for CTCF Xa- and both-preferred peaks. There was no significant CTCF co-localization with escape genes on the Xa in either brain or Patski cells. (C) Average CTCF Xi-SNP read counts in ten 100bp windows at promoters (0.5kb upstream and downstream of the TSS) is plotted against mRNA-seq Xi-SNP read counts escape genes (purple) and for genes subject to XCI (gray) in brain and Patski cells. In brain, a higher proportion of escape genes (6/14; Fisher’s exact test, p = 5e^-9^) had an average ≥10 reads (black line) at their promoter compared to genes subject to XCI (0/403). Similarly, in Patski cells a higher proportion of escape genes (9/65; Fisher’s exact test, p = 0.0004) had an average of ≥1 read (black line) at their promoter compared to genes subject to XCI (3/204).

**Fig 7:**
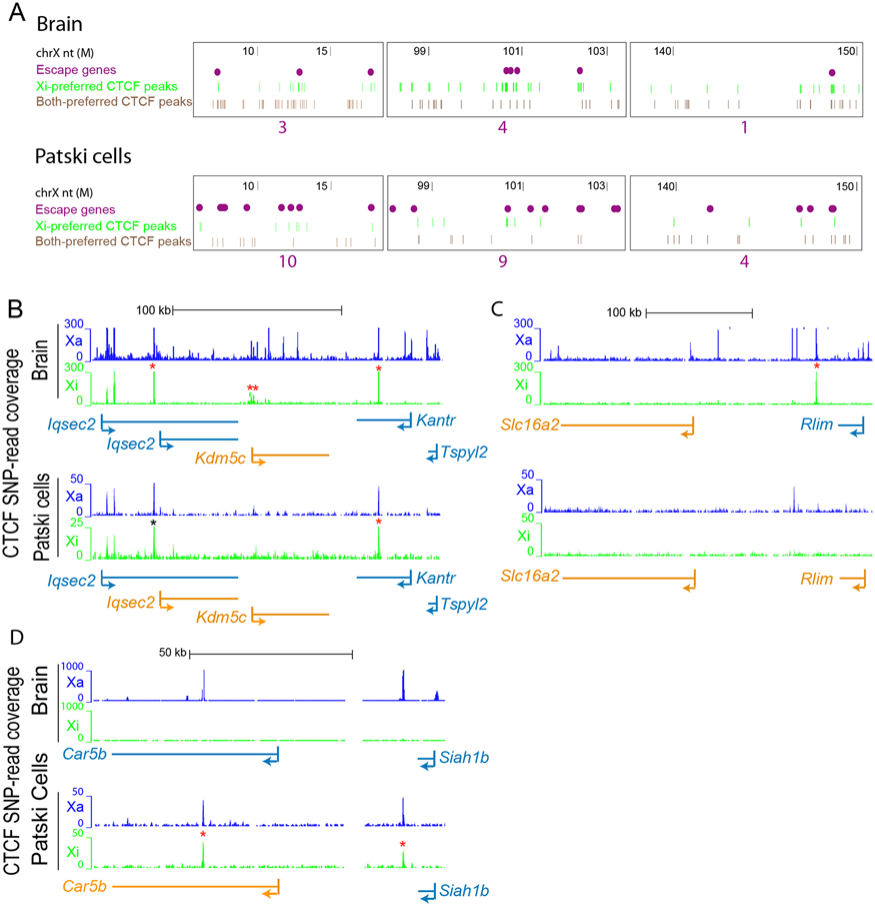
CTCF peaks density and distribution differ in brain and Patski cells. (A) Examples of Xi-preferred and both-preferred CTCF peaks distribution in three X chromosome regions (coordinates in million bp on top). The density of escape genes (purple dots, with total number under each region) is inversely related to the density of Xi-preferred (green) and both-preferred (brown) CTCF-binding peaks when comparing brain to Patski cells. (B) Allele-specific CTCF binding profiles around *Kdm5c*, a common escape gene flanked by *Iqsec2* and *Kantr*. In brain where only *Kdm5c* escapes XCI, CTCF binding is present at the 5’ end of the gene at the transition (double star) between *Kdm5c* and *Iqsec2* whose short and long transcripts are subject to XCI (see also S4A Fig). In Patski cells there is no such CTCF binding between *Kdm5c* and *Iqsec2*, which escapes XCI. CTCF also binds proximal of the *Iqsec2* short transcript in both brain and Patski cells, which could represent a proximal boundary of an escape domain. (C) Similar analysis at a region around *Rlim*, a gene that escapes XCI in Patski cells but not in brain, while the adjacent gene *Slc16a2* escapes XCI in both systems. A CTCF peak is present in the transition region only in brain. (D) Similar analysis in a region around *Car5b*, a gene that escapes XCI in Patski cells but not in brain (see also S4B Fig). CTCF binding peaks are located within the body of *Car5b* and in the transition between *Car5b* and *Siah1b* on the Xi in Patski cells. Genes that escape XCI are labeled orange and genes subject to XCI blue. Xa SNP reads are in blue and Xi SNP reads in green. Red stars indicate Xi- or both-preferred CTCF peaks on the Xi; one of the CTCF peaks is marked by a black star because it is present but was not called preferred at our cutoff.

To investigate the spatial distribution of Xi-binding CTCF peaks, the local Xi- and both-preferred CTCF peak density was calculated using a sliding window approach (window size: 500kb, step size: 1kb). Assuming that CTCF Xi-binding followed a Poisson distribution we fitted the data to estimate the Poisson parameters on the Xi, and calculated a p-value for each window. Enriched Xi-binding CTCF peaks were identified at a p-value cutoff of 0.01 and adjacent peaks were merged. Escape genes and Xi-binding CTCF clusters co-localized on the Xi but not the Xa, suggesting a role for CTCF binding at regions of escape (Fig. 6A and 6B). CTCF has been implicated in both transcription control and compartmentalization of the genome [48], thus, it was not surprising that CTCF peaks were found either at gene promoters or in intergenic regions. As expected, the 5'end of escape genes often displayed Xi-promoter occupancy by CTCF in both brain and Patski cells (Fig. 6C). To enrich in CTCF binding regions that might play a role in nuclear compartmentalization rather than transcription control, we then re-analyzed our data after excluding peaks located at promoters (±1kb from the TSS), which showed that CTCF clusters were still significantly associated with escape genes in brain (8/14 genes; p-value = 0.01, compared to a random sample of 500 X-linked genes; Fisher’s exact test) (S3B Fig). There was a similar trend in Patski cells, however the association (16/66 genes) was not significant, probably due to the lower number of CTCF peaks in the cell line. Interestingly, when allelic CTCF binding was analyzed in the context of higher order structure [43], CTCF Xi-preferred peaks were more significantly associated with 4C interacting domains than CTCF Xa-preferred peaks in brain and Patski cells (S8 Table). Furthermore, the density of CTCF peaks on the Xi was inversely related to the number of escape genes in brain and Patski cells as shown by inspecting three regions for the density of X-preferred and both-preferred CTCF binding peaks in relation to the number of escape genes (Fig. 7A). This is consistent with larger regions of escape in Patski cells (S6 Table).

We next examined allele-specific CTCF peak profiles in the UCSC genome browser at 200 and 139 regions of transitions between adjacent genes with a known XCI status in brain and Patski cells, respectively. Transitions were classified as between either two adjacent genes subject to XCI, an escape gene directly adjacent to a gene subject to XCI, or two adjacent escape genes (S9 Table). There were no transitions between two escape genes in brain, reflecting their low abundance compared to the Patski cell line in which 18 such transitions were observed. A larger proportion of transitions between genes with a different XCI status than those between two inactivated genes had CTCF peaks located in intergenic regions, 21% versus 15% in brain and 8% versus 3% in Patski cells, respectively (S9 Table). We then focused on specific transition regions: at the *Kdm5c-Iqsec2* region Xi-preferred CTCF binding peaks were found between *Iqsec2* and *Kdm5c* as well as within the gene body of *Kantr* located downstream of *Kdm5c* in brain where only *Kdm5c* escapes XCI (Figs. 7B and S4). In contrast, in Patski cells where *Kdm5c* and a short *Iqsec2* transcript both escape XCI, Xi-preferred CTCF binding peaks were found both upstream of the short *Iqsec2* transcript and within the gene body of *Kantr* but not between *Kdm5c* and *Iqsec2*, suggesting that lack of Xi-preferred CTCF binding in this region may contribute to a larger domain of escape in Patski cells (Fig. 7B). A similar situation was observed in the region between *Rlim* and *Slc16a2*, again suggesting that lack of CTCF binding in Patski cells led to a larger escape region (Fig. 7C). A different situation was seen at the *Car5b*-*Siah1b* region: Xi-preferred CTCF peaks were absent in brain where both *Car5b* and *Siah1b* are subject to XCI, while CTCF peaks flanked the *Car5b* promoter on both alleles in Patski cells where *Car5b* escapes XCI and *Siah1b* is subject to XCI (Figs. 7D and S4), suggesting that CTCF may play a role in the transition between escape and inactivated genes. Taken together, our results imply that CTCF binding may help configure escape domains via local chromatin looping, and/or facilitate the organization of escape genes at the periphery of the Xi territory.

## Discussion

Based on allele-specific analyses we identified genes expressed from both X chromosomes in female mouse tissues. Only a minority of these genes escape XCI in a tissue- and cell type-specific manner, indicating that XCI and escape from XCI are tightly controlled in vivo. The probability of bi-allelic versus mono-allelic expression was calculated using a new algorithm that can be applied to any gene in the genome. Our study represents the first comprehensive analysis of escape from XCI in vivo in multiple tissues.

**Fig 8:**
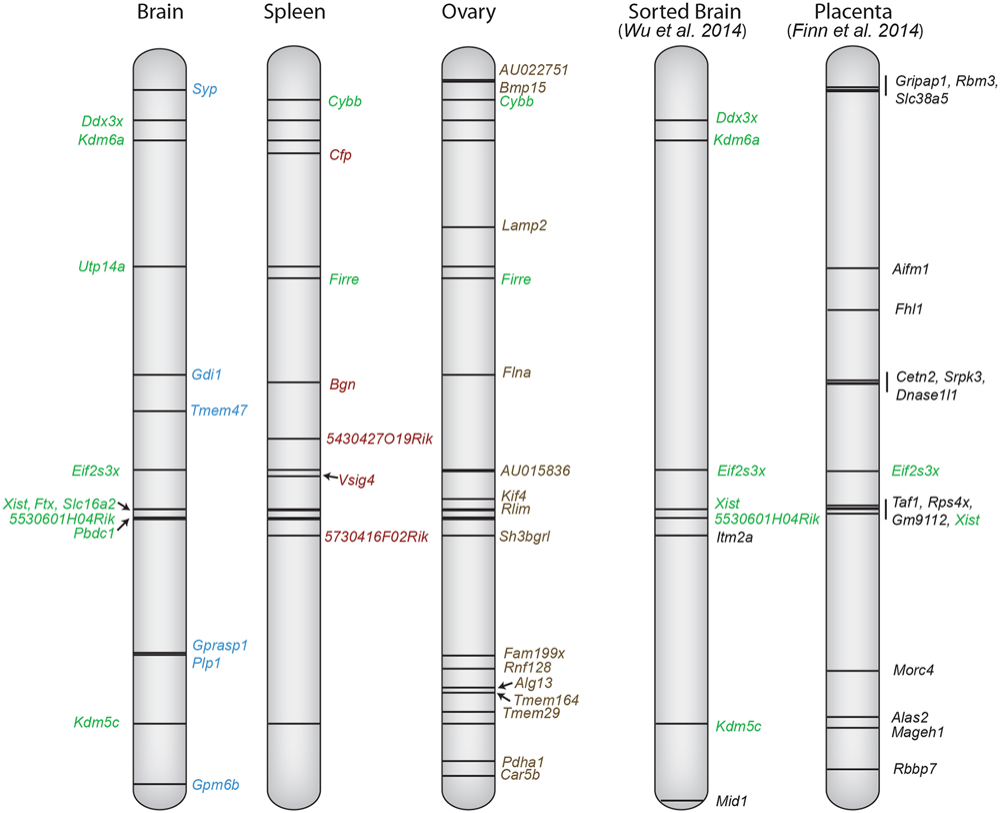
X chromosome escape maps differ between mouse tissues. The position of genes that escape XCI using a ≥2 Xi-SRPM cutoff (black lines) is shown at left for three tissues (brain, ovary and spleen) from F1 mice analyzed in our study. Gene names are color-coded to reflect their classification into group 1 (green, common in at least two tissues) or group 2 (blue, brain-specific escape; red, spleen-specific; brown, ovary-specific) based on our criteria (see Table 1). Coordinates at left are based on UCSC genome build NCBI37/mm9. For comparison, the genes reported to escape XCI in sorted brain cells from a *M*. *musculus* x *M*. *castaneus* cross [25], and genes reported to escape imprinted XCI in mid-gestation placenta from a *M*. *musculus* x *M*. *castaneus* cross [24] are shown at right. Genes labeled green are common between studies.

Our data and those of others indicate that for a subset of X-linked genes, escape from XCI is ubiquitous and thus represents an intrinsic property of these genes [22,43]. Among these common escape genes *Ddx3x*, *Kdm6a*, *Eif2s3x* and *Kdm5c* represent genes that each has a conserved Y-linked paralog with a similar function [49,50]. These X/Y genes play important roles in the regulation of transcription and translation and are highly dosage-sensitive, which could explain why they consistently escape XCI in all tissues examined (Fig. 8). Interestingly, we also identified tissue-specific escape genes, which will help understanding of functional mechanisms leading to sex differences in these tissues. For example, we identified six genes that escape XCI in brain, all of which have been implicated in brain functions, *Gpm6b*, *Gprasp1*, *Syp*, *Gdi1*, *Plp1*, and *Tmem47* [51]. Our results generally agree with a recent study of XCI based on flow-sorted brain cells with differentially labeled BL6 and *Mus castaneus* X chromosomes [25] (Fig. 8). Of seven escape genes reported in that study, five are included in the present study (*5530601H04Rik*, *Ddx3x*, *Eif2s3x*, *Kdm5c*, and *Kdm6a*). The two other genes are *Itm2a* that had too few Xi reads to be classified in our study, and *Mid1* we have shown to be subject to XCI in *M*. *spretus* where it is outside the PAR (this study), while it escapes XCI in BL6 mouse brain [31,52].

Importantly, many of the escape genes we identified have significant female sex bias in expression, suggesting roles in sex differences (S5 Table) [1,30,53–57]. For example, three of the brain-specific escape genes we identified in mouse, *GPM6B*, *SYP*, and *PLP1* also escape XCI in human, resulting in higher expression in female than male brain [6,58]. Whether deficiency in these genes due to the presence of a single X chromosome in women with Turner syndrome contributes to mild cognitive impairment remains to be determined [59]. Interestingly, of the five novel spleen-specific escape genes we identified, as many as three, *Vsig4*, *Cfp*, and *Bgn*, have been implicated in autoimmune disorders both in mouse and human [36,38,39]. It is well established that autoimmune disorders are much more common in women and their incidence is increased in Turner syndrome, but specific genetic mechanisms are not well defined [60]. Our in vivo study demonstrates that analyses of relevant human tissues, for example spleen in the case of *CFP* and *BGN*, two gene previously classified as being subject to XCI in cell cultures [6], will be critical to understand sex differences in specific disorders.

Do genes escape XCI only in tissues where they are most highly expressed? While genes that escape XCI often have high expression in a particular tissue, expression does not appear to be the sole driving force for escape. For example, *Car5b* is more highly expressed in ovary (42RPKM) than in Patski cells (8RPKM) and yet escapes XCI only in Patski cells. A previous study identified a set of 17 escape genes in mouse placenta [24] (Fig. 8). Many of these differ from escape genes found in our study, probably because extra-embryonic tissues undergo imprinted paternal XCI, which differs from random XCI [61]. A comprehensive comparison of escape from XCI in available mouse tissues shows that only *Eif2s3x* escapes XCI in all tissues examined (Fig. 8). We found that escape from XCI represents a continuum of expression from the Xi compared to the Xa. While our data only includes genes expressed above a strict cutoff of 1RPKM, we cannot exclude that some genes with lower expression may also escape XCI. For genes with significant expression from the Xi (excluding *Xist*), expression ranged from 3–105% (median 18%) of the Xa expression level. Thus, expression from the Xi was usually lower than that of the Xa in mouse, similar to what has been reported in human, even though there are more escape genes in this species based on expression analyses of cell cultures [6] and on DNA methylation profiles in human tissues where 9% of human genes were found to have a methylation pattern consistent with escape [62]. The mouse with 3–7% escape genes in tissues may be exceptional compared to other mammals in which XCI patterns are often more similar to the human pattern [3,63].

Our findings of a larger number of escape genes in Patski cells compared to mouse tissues may reflect either the acquisition of epigenetic changes leading to reactivation of X-linked genes in cell culture or a genuine property of these cells. Our analyses suggest that kidney-specific escape genes do exist and could explain in part the pattern seen in Patski cells, but we also found significant differences between the cell line and the tissue of origin. Clustering of escape genes in Patski cells but not in tissues suggests unstable silencing of large chromosomal regions. Using an in vitro system of cultured trophoblastic cells Calabrese et al. also identified a relatively large number of escape genes (35 out of 262 accessible) [14], which represents twice the number of escape genes found in mouse placenta [24]. Furthermore, 22/66 escape genes in Patski cells are in regions of escape reported in cultured neural progenitor cells [43]. Thus, the number of escape genes may be overestimated when based on studies of cultured cells, which are notoriously susceptible to epigenetic changes such as DNA methylation changes associated with gene expression and CTCF binding aberrations [64,65]. We cannot rule out the possibility that XCI in embryonic cells including embryonic kidney cells from which Patski cells were derived, as well as trophoblastic cells and neural progenitor cells may simply be less complete than in adult tissues. Future studies will help sort out developmental aspects of escape from XCI.

We found a good correlation between escape from XCI and regulatory features associated with transcription, such as PolII-S5p occupancy and DNase I hypersensitivity at the promoters of genes on the Xi, indicating that escape regions have a more open chromatin configuration. This is consistent with escape genes being associated with histone marks characteristic of active chromatin [13–18,66]. Interestingly, we found distinct CTCF binding patterns on the Xi and Xa. A study in human cells also reported that while a majority of CTCF peaks on the X chromosome are bi-allelic, some peaks are Xa- or Xi-specific [67]. In addition, a recent study in differentiated mouse ES cells also describes significant differences between Xa- and Xi-specific CTCF peaks at escape gene loci (determined by ChIP-seq), as well as differences in interactions between transcripts and CTCF (determined by CLIP-seq) [68]. These findings are in contrast to a study of imprinted XCI, in which Xi and Xa CTCF binding patterns were nearly identical [14]. Thus, the role of CTCF in escape from XCI may differ between random and imprinted XCI. CTCF binding peaks were often located at the promoters of genes expressed from the Xi, in agreement with a role for CTCF in transcription regulation [69]. In addition, since CTCF binding peaks located in intergenic regions also clustered with escape genes, CTCF may also be a factor in compartmentalization of the Xi. Chromatin interactions such as looping as determined by Hi-C are correlated with the distribution of CTCF binding [70]. This is supported by our findings that Xi-preferred CTCF binding is more significantly associated with 4C interacting domains. Indeed, CTCF plays an important role in nuclear structure and is often found at the boundary between topological domains [71–73]. Furthermore, regions containing escape genes are preferentially engaged in long range *cis*-interactions [43]. Previous studies have shown that specific boundary elements possibly involving CTCF may have a role in the segregation of silenced domains from escape domains [21,74]. The low density of CTCF peaks observed in Patski cells may result in a more relaxed structure of the Xi in the cell line, leading to an expansion of escape domains. Interestingly, disruption of CTCF binding at the borders of domains enriched in H3K27me3 in *Drosophila* results in a reduction in H3K27me3 levels in repressed domains [75], and loss of CTCF binding at super-enhancers results in increased expression of adjacent genes [76]. It is important to note that a previous 5C study of the XIC has reported CTCF binding both at the boundaries of topologically associating domains (TADs) and within TADs, suggesting that CTCF is not the sole factor in determining Xi organization [77]. Further studies will help define other elements that may help structure the Xi.

In summary, we demonstrate the utility of a mouse model to study XCI in vivo. Using this resource novel tissue-specific escape genes have been identified. Escape genes are associated with an open chromatin structure and CTCF binding may influence the definition of differential chromatin architecture of the X.

## Materials and Methods

### Tissue collection, hybrid mouse model and cell culture

Ovaries, spleen, liver, and whole brain were collected from female F1 obtained by mating C57B/6J females that carry a deletion of the *Xist* proximal A-repeat (*Xist* ^*Δ*^) (B6.Cg-Xist<tm5Sado>, RIKEN) [27] with *M*. *spretus* males (Jackson Labs). Female progeny were genotyped to verify inheritance of the *Xist* ^Δ^ allele using specific primers [27]. F1 mice that inherited a maternal X chromosome with an *Xist* ^*Δ*^ fail to silence the BL6 X and thus have complete skewing of XCI of the paternal *spretus* X. All procedures involving animals were reviewed and approved by the University Institutional Animal Care and Use Committee (IACUC), and were performed in accordance with the Guiding Principles for the Care and Use of Laboratory Animals. Patski cells were cultured as previously described [18].

### Validation of allelic expression

To verify skewing of XCI, cDNA and control genomic DNA (gDNA) extracted from each tissue were subject to PCR amplification of *Ubqln2* followed by Sanger sequencing (S10 Table). A similar approach was used to confirm the XCI status of *Mid1*, *Bmp15* and *Vsig4*, *Rlim*, *Shroom4*, *Car5b*, and *5530601H04Rik* (S10 Table). Allele-specific RT-PCR was done to confirm Xi expression of *Plp1*, *Cfp*, *Hdac6*. Briefly, cDNA was made by Superscript II reverse transcriptase (Life Technologies) using oligo-dT primers according to manufacturer's protocol. PCR reactions with non-species specific and BL6-specific or *spretus*-specific primers (S10 Table) were performed using tissues from BL6, *spretus* and *Xist* ^*Δ*^ hybrid F1 mice. *Actinβ* was used as a positive control. For quantification, gel band intensities were measured using ImageJ software (http://imagej.nih.gov/ij/) and, together with RNA-seq Xi levels, plotted to compare expression from the Xi and Xa.

### ChIP-seq with allele-specific analyses

ChIP-seq using PolII-S5p (Abcam) and CTCF (Millipore) ChIP-grade antibodies were performed as described [26]. The specificity of the PolII-S5p antibody (Abcam) was verified by blocking immunostaining with synthetic peptides (Abcam ab18488). A pseudo-*spretus* genome was assembled by substituting available SNPs (from Sanger) into the BL6 UCSC Genome Browser NCBIv37/mm9 reference genome. Reads from genomic DNA sequencing, ChIP-seq, and DNase I-seq experiments were mapped separately to the BL6 reference sequence (mm9) and to the pseudo-*spretus* genome using BWA/v0.5.9 [78] with default parameters. Only those reads that mapped uniquely and with a high-quality mapping score (MAPQ ≥ 30) to either the BL6 genome or the pseudo-*spretus* genome were kept for allele-specific analyses (see details in main text).

### RNA-seq with allele-specific analyses

RNA-seq experiments were done as described [18,26]. Exonic RNA-seq reads were mapped using bowtie/v0.12.7 [79] to both the genome and transcriptome and gene expression was estimated using Tophat/v2.0.2 [29] with default parameters. Only those reads that mapped uniquely and with a high-quality mapping score (MAPQ ≥ 30) to either the BL6 genome or the pseudo-*spretus* genome were kept for allele-specific analyses. Since *Eif2s3x* exons (except exon 1) have a high sequence similarity to another X region (chrX: 31680780–31684279), we included reads contained in exons with a low MAPQ score for this gene. Post filtering of expression levels and Xi SNP reads was done to remove genes with low expression and/or limited Xi-SNP reads. In addition, a binomial model for comparison of Xi-SNP reads to total-SNP reads (Xi+Xa) for all exons of each gene was used to call genes that escaped at levels below the Xi/Xa ratio threshold cutoff. Reads containing informative SNPs were assigned to each haploid genome. For the whole X chromosome we used 1,532,011 SNPs, including 597,315 SNPs in gene bodies, and 31,062 SNPs in exons. Gene expression analyses were performed as described in the main text.

### Data access

RNA-seq data for the Patski cell line are deposited to the NCBI Gene expression Omnibus (GEO; http://www.ncbi.nlm.nih.gov/geo/) under accession number GSM970866. ChIP-seq data for PolII-S5p occupancy in brain and Patski cells are deposited under the accession number GSE44255. RNA-seq data for mouse tissues and ChIP-seq data for CTCF binding in brain and Patski cells is deposited to the GEO database under accession number GSE59779. Patski cell line DNase I hypersensitivity data is deposited under the accession number GSM1014171.

### Ethics statement

For mice sacrificed, euthanasia was accomplished using two methods (carbon dioxide asphyxiation followed by cervical dislocation) as required by the University of Washington's Office of Animal Welfare. Husbandry and all other procedures were approved by University of Washington's Office of Animal Welfare.

## Supporting Information

S1 Fig Validation of skewing of XCI in mouse model and validation of *Plp1*, *Mid1*, *Vsig4*, and *Bmp15* expression profiles. (A) Sanger sequencing of *Ubqln2* RT-PCR products confirms XCI skewing in F1 female mice. cDNA tracings show only the BL6 allele in brain, ovary, and spleen, while gDNA tracing confirms heterozygosity at a SNP (C in BL6 and T in *spretus*). Arrows indicate SNP positions. (B) mRNA SNP read distribution profiles obtained by RNA-seq for *Ubqln2* demonstrate the absence of *spretus* Xi reads in brain, ovary and spleen. Xa SNP reads are in blue and Xi SNP reads in green. (C) Validation of escape from XCI for *Plp1* using RT-PCR with species-specific primers. Gel electrophoresis of RT-PCR products using non-species-specific primers and *spretus*-specific primers (S10 Table) in BL6, *spretus*, and F1 brain in which the Xi is from *spretus*. *ActinB* was used as a control. Control reactions include "No RT" (no reverse transcriptase) and H_2_O (instead of primers). (D) Xi expression of *Plp1* was determined to be 1.5% of total *Plp1* expression in F1 brain by gel band quantification measured by Imagej. (E) *Mid1* cDNA Sanger sequencing confirms inactivation of the *spretus* allele in brain, while gDNA tracing shows heterozygosity of *Mid1* (C in BL6 and G *in spretus*). Arrows indicate SNP positions. (F) mRNA SNP read distribution profiles obtained by RNA-seq for *Vsig4* a gene that escapes XCI in spleen, but is subject to XCI in liver. Xa SNP reads are in blue and Xi SNP reads in green. (G) *Vsig4* cDNA Sanger sequencing tracings confirm bi-allelic expression in spleen but not liver, while gDNA tracings show SNP heterozygosity (T in BL6 and C in *spretus*). Arrows indicate SNP positions. (H) mRNA SNP read distribution profiles obtained by RNA-seq show bi-allelic expression of *Bmp15* in ovary, but not in brain or spleen. Xa SNP reads are in blue and Xi SNP reads in green. (I) *Bmp15* cDNA Sanger sequencing tracing confirms escape from XCI for in ovary while gDNA tracing shows SNP heterozygosity (A in BL6 and G in *spretus*). Arrows indicate SNP positions.
(PDF)

S2 Fig PolII-S5p enrichment, DNase I sensitivity correlate with escape from XCI. (A, B) Examples of allele-specific PolII-S5p occupancy profiles and expression (mRNA) profiles at *Kdm6a*, a common escape gene in brain (A) and Patski cells (B). PolII-S5p is enriched at the promoter region (highlighted by a red box) on both the Xa and the Xi. DNase I hypersensitivity tested in Patski cells only is also increased at the promoter region (highlighted by a red box) on both the Xa and Xi. Xa SNP reads are in blue and Xi SNP reads in green. (C, D) Same analysis for the lncRNA *5530601H04Rik*, another common escape gene. (E, F) Same analysis for *Shroom4*, a gene subject to XCI in brain (labeled blue) but that escapes XCI in Patski cells (labeled orange). PolII-S5p is enriched at the promoter region (highlighted by a red box) of *Shroom4* on both the Xa and the Xi in Patski cells, whereas enrichment is limited to the Xa in brain. DNase I hypersensitivity tested in Patski cells only is also increased at the promoter region (highlighted by a red box) on both the Xa and Xi.
(PDF)

S3 Fig Verification of CTCF SNP-reads at imprinted autosomal genes and distribution of non-promoter CTCF binding on the Xi. (A) CTCF ChIP-seq analysis in brain and Patski cells at two imprinted regions. On mouse chromosome 7 *H19* is only expressed from the maternal allele while *Peg13* on mouse chromosome 15 is expressed from the paternal allele. CTCF binding upstream of these genes is high on the allele from which they are expressed, in agreement with a previous study [44]. M, maternal allele and P, paternal allele, T, total reads from both alleles. The differentially methylated regions (DMR) are indicated. (B) Non-promoter significant CTCF Xi-binding clusters were mapped along the Xi in brain and Patski cells (compare to Fig. 6A). After CTCF peaks located around promoters (±1kb from the TSS) were excluded Xi- and both-preferred peaks were determined by a binomial model and used for density analysis. Red bars represent merger of clusters of CTCF Xi-binding peaks, while purple dots represent escape genes. Non-promoter significant Xi-binding CTCF binding clusters tend to co-localize in regions containing escape genes and are more abundant in brain than Patski cells. Horizontal axis represents the Xi in Mb. The vertical axis is the negative log of the calculated binomial p-value [-log (p-value)]. The thin red dashed line represents a 0.01 p-value cutoff.
(PDF)

S4 Fig SNP-reads distribution for mRNA and CTCF. (A) Example of mRNA SNP read distribution profiles and allele-specific CTCF distribution profiles at the *Kdm5c*-*Iqsec2* region in brain and Patski cells (see also Fig. 7). (B) Example of mRNA SNP read distribution profiles and allele-specific CTCF distribution profiles at the *Car5b* and *Siah1b* region in brain and Patski cells (see also Fig. 7). RNA-seq read quantification was done by normalizing reads from the Xi to total reads (Xi + Xa) in two biological replicates. Xa SNP reads are in blue and Xi SNP reads in green. Genes that escape XCI are labeled orange and genes subject to XCI blue.
(PDF)

S1 Table Summary of X-linked genes examined by allelic RNA-seq expression analysis in mouse tissues and Patski cells. (XLSX)

S2 Table Escape genes in brain using ≥2 Xi-SRPM cutoff. (XLSX)

S3 Table Escape genes in spleen using ≥2 Xi-SRPM cutoff. (XLSX)

S4 Table Escape genes in ovary using ≥2 Xi-SRPM cutoff. (XLSX)

S5 Table Functions of genes that escape XCI. (XLSX)

S6 Table Domain distribution of escape genes in Patski cells using ≥2 Xi-SRPM cutoff. (XLSX)

S7 Table CTCF binding peaks located on the Xi or on both Xi and Xa in brain and Patski cells. (XLSX)

S8 Table Analysis of CTCF allelic enrichment in 4C domains in brain and Patski cells (XLSX)

S9 Table Analysis of CTCF peaks in transition regions (XLSX)

S10 Table Primers for Sanger sequencing and allelic validation. (XLSX)

S1 Dataset RPKM and RNA-SNP-read counts in Patski cells and tissues. (XLS)

S2 Dataset Allelic X-promoter association of PolII and CTCF in brain. (XLSX)

S3 Dataset Allelic X-promoter association of PolII and CTCF in Patski cells. (XLSX)

## References

[1] Deng X, Berletch JB, Nguyen DK, Disteche CM (2014) X chromosome regulation: diverse patterns in development, tissues and disease. Nat Rev Genet 15: 367–378. 10.1038/nrg3687 24733023 PMC4117651

[2] Lessing D, Anguera MC, Lee JT (2013) X chromosome inactivation and epigenetic responses to cellular reprogramming. Annu Rev Genomics Hum Genet 14: 85–110. 10.1146/annurev-genom-091212-153530 23662665

[3] Berletch JB, Yang F, Xu J, Carrel L, Disteche CM (2011) Genes that escape from X inactivation. Hum Genet 130: 237–245. 10.1007/s00439-011-1011-z 21614513 PMC3136209

[4] Peeters SB, Cotton AM, Brown CJ (2014) Variable escape from X-chromosome inactivation: Identifying factors that tip the scales towards expression. Bioessays 10.1002/bies.201400032 PMC4143967 24913292

[5] Anderson CL, Brown CJ (2002) Variability of X chromosome inactivation: effect on levels of TIMP1 RNA and role of DNA methylation. Hum Genet 110: 271–278. 11935340 10.1007/s00439-002-0676-8

[6] Carrel L, Willard HF (2005) X-inactivation profile reveals extensive variability in X-linked gene expression in females. Nature 434: 400–404. 15772666 10.1038/nature03479

[7] Cotton AM, Ge B, Light N, Adoue V, Pastinen T, et al (2013) Analysis of expressed SNPs identifies variable extents of expression from the human inactive X chromosome. Genome Biol 14: R122 10.1186/gb-2013-14-11-r122 24176135 PMC4053723

[8] Bondy CA (2007) Care of girls and women with Turner syndrome: a guideline of the Turner Syndrome Study Group. J Clin Endocrinol Metab 92: 10–25. 17047017 10.1210/jc.2006-1374

[9] Zinn AR, Page DC, Fisher EM (1993) Turner syndrome: the case of the missing sex chromosome. Trends Genet 9: 90–93. 8488568 10.1016/0168-9525(93)90230-f

[10] Murakami K, Ohhira T, Oshiro E, Qi D, Oshimura M, et al (2009) Identification of the chromatin regions coated by non-coding Xist RNA. Cytogenet Genome Res 125: 19–25. 10.1159/000207514 19617692

[11] Engreitz JM, Pandya-Jones A, McDonel P, Shishkin A, Sirokman K, et al (2013) The Xist lncRNA exploits three-dimensional genome architecture to spread across the X chromosome. Science 341: 1237973 10.1126/science.1237973 23828888 PMC3778663

[12] Simon MD, Pinter SF, Fang R, Sarma K, Rutenberg-Schoenberg M, et al (2013) High-resolution Xist binding maps reveal two-step spreading during X-chromosome inactivation. Nature 504: 465–469. 10.1038/nature12719 24162848 PMC3904790

[13] Boggs BA, Cheung P, Heard E, Spector DL, Chinault AC, et al (2002) Differentially methylated forms of histone H3 show unique association patterns with inactive human X chromosomes. Nat Genet 30: 73–76. 11740495 10.1038/ng787

[14] Calabrese JM, Sun W, Song L, Mugford JW, Williams L, et al (2012) Site-specific silencing of regulatory elements as a mechanism of X inactivation. Cell 151: 951–963. 10.1016/j.cell.2012.10.037 23178118 PMC3511858

[15] Changolkar LN, Singh G, Cui K, Berletch JB, Zhao K, et al (2010) Genome-wide distribution of macroH2A1 histone variants in mouse liver chromatin. Mol Cell Biol 30: 5473–5483. 10.1128/MCB.00518-10 20937776 PMC2976432

[16] Gilbert SL, Sharp PA (1999) Promoter-specific hypoacetylation of X-inactivated genes. Proc Natl Acad Sci U S A 96: 13825–13830. 10570157 10.1073/pnas.96.24.13825 PMC24149

[17] Khalil AM, Driscoll DJ (2007) Trimethylation of histone H3 lysine 4 is an epigenetic mark at regions escaping mammalian X inactivation. Epigenetics 2: 114–118. 17965609 10.4161/epi.2.2.4612

[18] Yang F, Babak T, Shendure J, Disteche CM (2010) Global survey of escape from X inactivation by RNA-sequencing in mouse. Genome Res 20: 614–622. 10.1101/gr.103200.109 20363980 PMC2860163

[19] Cotton AM, Lam L, Affleck JG, Wilson IM, Penaherrera MS, et al (2011) Chromosome-wide DNA methylation analysis predicts human tissue-specific X inactivation. Hum Genet 130: 187–201. 10.1007/s00439-011-1007-8 21597963 PMC3132437

[20] Lister R, Mukamel EA, Nery JR, Urich M, Puddifoot CA, et al (2013) Global epigenomic reconfiguration during mammalian brain development. Science 341: 1237905 10.1126/science.1237905 23828890 PMC3785061

[21] Filippova GN, Cheng MK, Moore JM, Truong JP, Hu YJ, et al (2005) Boundaries between chromosomal domains of X inactivation and escape bind CTCF and lack CpG methylation during early development. Dev Cell 8: 31–42. 15669143 10.1016/j.devcel.2004.10.018

[22] Li N, Carrel L (2008) Escape from X chromosome inactivation is an intrinsic property of the Jarid1c locus. Proc Natl Acad Sci U S A 105: 17055–17060. 10.1073/pnas.0807765105 18971342 PMC2579377

[23] Lingenfelter PA, Adler DA, Poslinski D, Thomas S, Elliott RW, et al (1998) Escape from X inactivation of Smcx is preceded by silencing during mouse development. Nat Genet 18: 212–213. 9500539 10.1038/ng0398-212

[24] Finn EH, Smith CL, Rodriguez J, Sidow A, Baker JC (2014) Maternal bias and escape from X chromosome imprinting in the midgestation mouse placenta. Dev Biol 390: 80–92. 10.1016/j.ydbio.2014.02.020 24594094 PMC4045483

[25] Wu H, Luo J, Yu H, Rattner A, Mo A, et al (2014) Cellular resolution maps of x chromosome inactivation: implications for neural development, function, and disease. Neuron 81: 103–119. 10.1016/j.neuron.2013.10.051 24411735 PMC3950970

[26] Deng X, Berletch JB, Ma W, Nguyen DK, Hiatt JB, et al (2013) Mammalian X Upregulation Is Associated with Enhanced Transcription Initiation, RNA Half-Life, and MOF-Mediated H4K16 Acetylation. Dev Cell S1534–5807(13)00101–9 [pii] 10.1016/j.devcel.2013.01.028. 10.1016/j.devcel.2013.01.028 PMC3662796 23523075

[27] Hoki Y, Kimura N, Kanbayashi M, Amakawa Y, Ohhata T, et al (2009) A proximal conserved repeat in the Xist gene is essential as a genomic element for X-inactivation in mouse. Development 136: 139–146. 10.1242/dev.026427 19036803

[28] Waterston RH, Lindblad-Toh K, Birney E, Rogers J, Abril JF, et al (2002) Initial sequencing and comparative analysis of the mouse genome. Nature 420: 520–562. 12466850 10.1038/nature01262

[29] Trapnell C, Williams BA, Pertea G, Mortazavi A, Kwan G, et al (2010) Transcript assembly and quantification by RNA-Seq reveals unannotated transcripts and isoform switching during cell differentiation. Nat Biotechnol 28: 511–515. 10.1038/nbt.1621 20436464 PMC3146043

[30] Reinius B, Johansson MM, Radomska KJ, Morrow EH, Pandey GK, et al (2012) Abundance of female-biased and paucity of male-biased somatically expressed genes on the mouse X-chromosome. BMC Genomics 13: 607 10.1186/1471-2164-13-607 23140559 PMC3534601

[31] Nguyen DK, Yang F, Kaul R, Alkan C, Antonellis A, et al (2011) Clcn4–2 genomic structure differs between the X locus in Mus spretus and the autosomal locus in Mus musculus: AT motif enrichment on the X. Genome Res 21: 402–409. 10.1101/gr.108563.110 21282478 PMC3044854

[32] Werner HB, Kramer-Albers EM, Strenzke N, Saher G, Tenzer S, et al (2013) A critical role for the cholesterol-associated proteolipids PLP and M6B in myelination of the central nervous system. Glia 61: 567–586. 10.1002/glia.22456 23322581

[33] Gordon SL, Cousin MA (2013) X-linked intellectual disability-associated mutations in synaptophysin disrupt synaptobrevin II retrieval. J Neurosci 33: 13695–13700. 10.1523/JNEUROSCI.0636-13.2013 23966691 PMC3755716

[34] Strobl-Wildemann G, Kalscheuer VM, Hu H, Wrogemann K, Ropers HH, et al (2011) Novel GDI1 mutation in a large family with nonsyndromic X-linked intellectual disability. Am J Med Genet A 155A: 3067–3070. 10.1002/ajmg.a.34291 22002931

[35] Mathis C, Bott JB, Candusso MP, Simonin F, Cassel JC (2011) Impaired striatum-dependent behavior in GASP-1-knock-out mice. Genes Brain Behav 10: 299–308. 10.1111/j.1601-183X.2010.00666.x 21091868

[36] Choi HM, Lee YA, Yang HI, Yoo MC, Kim KS (2011) Increased levels of thymosin beta4 in synovial fluid of patients with rheumatoid arthritis: association of thymosin beta4 with other factors that are involved in inflammation and bone erosion in joints. Int J Rheum Dis 14: 320–324. 10.1111/j.1756-185X.2011.01652.x 22004227

[37] Kim KH, Choi BK, Song KM, Cha KW, Kim YH, et al (2013) CRIg signals induce anti-intracellular bacterial phagosome activity in a chloride intracellular channel 3-dependent manner. Eur J Immunol 43: 667–678. 10.1002/eji.201242997 23280470

[38] Lesher AM, Nilsson B, Song WC (2013) Properdin in complement activation and tissue injury. Mol Immunol 56: 191–198. 10.1016/j.molimm.2013.06.002 23816404 PMC3730815

[39] Moreth K, Brodbeck R, Babelova A, Gretz N, Spieker T, et al (2010) The proteoglycan biglycan regulates expression of the B cell chemoattractant CXCL13 and aggravates murine lupus nephritis. J Clin Invest 120: 4251–4272. 10.1172/JCI42213 21084753 PMC2993585

[40] Heard E, Turner J (2011) Function of the sex chromosomes in mammalian fertility. Cold Spring Harbor perspectives in biology 3: a002675 10.1101/cshperspect.a002675 21730045 PMC3179336

[41] ENCODE Project Consortium MR, Stamatoyannopoulos J, Snyder M, Dunham I, Hardison RC, Bernstein BE, Gingeras TR, Kent WJ, Birney E et al (2011) A user's guide to the encyclopedia of DNA elements (ENCODE). PLoS Biol 9: e1001046 10.1371/journal.pbio.1001046 21526222 PMC3079585

[42] Hacisuleyman E, Goff LA, Trapnell C, Williams A, Henao-Mejia J, et al (2014) Topological organization of multichromosomal regions by the long intergenic noncoding RNA Firre. Nat Struct Mol Biol 21: 198–206. 10.1038/nsmb.2764 24463464 PMC3950333

[43] Splinter E, de Wit E, Nora EP, Klous P, van de Werken HJ, et al (2011) The inactive X chromosome adopts a unique three-dimensional conformation that is dependent on Xist RNA. Genes Dev 25: 1371–1383. 10.1101/gad.633311 21690198 PMC3134081

[44] Prickett AR, Barkas N, McCole RB, Hughes S, Amante SM, et al (2013) Genomewide and parental allele-specific analysis of CTCF and cohesin DNA binding in mouse brain reveals a tissue-specific binding pattern and an association with imprinted differentially methylated regions. Genome Res gr.150136.112 [pii] 10.1101/gr.150136.112. 10.1101/gr.150136.112 PMC3787260 23804403

[45] Ji H, Jiang H, Ma W, Johnson DS, Myers RM, et al (2008) An integrated software system for analyzing ChIP-chip and ChIP-seq data. Nat Biotechnol 26: 1293–1300. 10.1038/nbt.1505 18978777 PMC2596672

[46] Ma W, Wong WH (2011) The analysis of ChIP-Seq data. Methods Enzymol 497: 51–73. 10.1016/B978-0-12-385075-1.00003-2 21601082

[47] Zhang Y, Liu T, Meyer CA, Eeckhoute J, Johnson DS, et al (2008) Model-based analysis of ChIP-Seq (MACS). Genome Biol 9: R137 10.1186/gb-2008-9-9-r137 18798982 PMC2592715

[48] Ong CT, Corces VG (2014) CTCF: an architectural protein bridging genome topology and function. Nat Rev Genet 15: 234–246. 10.1038/nrg3663 24614316 PMC4610363

[49] Bellott DW, Hughes JF, Skaletsky H, Brown LG, Pyntikova T, et al (2014) Mammalian Y chromosomes retain widely expressed dosage-sensitive regulators. Nature 508: 494–499. 10.1038/nature13206 24759411 PMC4139287

[50] Cortez D, Marin R, Toledo-Flores D, Froidevaux L, Liechti A, et al (2014) Origins and functional evolution of Y chromosomes across mammals. Nature 508: 488–493. 10.1038/nature13151 24759410

[51] Utami KH, Hillmer AM, Aksoy I, Chew EG, Teo AS, et al (2014) Detection of chromosomal breakpoints in patients with developmental delay and speech disorders. PLoS One 9: e90852 10.1371/journal.pone.0090852 24603971 PMC3946304

[52] Dal Zotto L, Quaderi NA, Elliott R, Lingerfelter PA, Carrel L, et al (1998) The mouse Mid1 gene: implications for the pathogenesis of Opitz syndrome and the evolution of the mammalian pseudoautosomal region. Hum Mol Genet 7: 489–499. 9467009 10.1093/hmg/7.3.489

[53] Isensee J, Witt H, Pregla R, Hetzer R, Regitz-Zagrosek V, et al (2008) Sexually dimorphic gene expression in the heart of mice and men. J Mol Med (Berl) 86: 61–74. 17646949 10.1007/s00109-007-0240-z PMC2755745

[54] Li J, Chen X, McClusky R, Ruiz-Sundstrom M, Itoh Y, et al (2014) The number of X chromosomes influences protection from cardiac ischaemia/reperfusion injury in mice: one X is better than two. Cardiovasc Res 102: 375–384. 10.1093/cvr/cvu064 24654234 PMC4030514

[55] Xu J, Burgoyne PS, Arnold AP (2002) Sex differences in sex chromosome gene expression in mouse brain. Hum Mol Genet 11: 1409–1419. 12023983 10.1093/hmg/11.12.1409

[56] Xu J, Deng X, Disteche CM (2008) Sex-specific expression of the X-linked histone demethylase gene Jarid1c in brain. PLoS One 3: e2553 10.1371/journal.pone.0002553 18596936 PMC2438472

[57] Xu J, Deng X, Watkins R, Disteche CM (2008) Sex-specific differences in expression of histone demethylases Utx and Uty in mouse brain and neurons. J Neurosci 28: 4521–4527. 10.1523/JNEUROSCI.5382-07.2008 18434530 PMC2643472

[58] Brawand D, Soumillon M, Necsulea A, Julien P, Csardi G, et al (2011) The evolution of gene expression levels in mammalian organs. Nature 478: 343–348. 10.1038/nature10532 22012392

[59] Knickmeyer RC (2012) Turner syndrome: advances in understanding altered cognition, brain structure and function. Curr Opin Neurol 25: 144–149. 10.1097/WCO.0b013e3283515e9e 22322416

[60] Jorgensen KT, Rostgaard K, Bache I, Biggar RJ, Nielsen NM, et al (2010) Autoimmune diseases in women with Turner's syndrome. Arthritis Rheum 62: 658–666. 10.1002/art.27270 20187158

[61] Takagi N, Sasaki M (1975) Preferential inactivation of the paternally derived X chromosome in the extraembryonic membranes of the mouse. Nature 256: 640–642. 1152998 10.1038/256640a0

[62] Cotton AM, Chen CY, Lam LL, Wasserman WW, Kobor MS, et al (2014) Spread of X-chromosome inactivation into autosomal sequences: role for DNA elements, chromatin features and chromosomal domains. Hum Mol Genet 23: 1211–1223. 10.1093/hmg/ddt513 24158853 PMC4051349

[63] Al Nadaf S, Deakin JE, Gilbert C, Robinson TJ, Graves JA, et al (2012) A cross-species comparison of escape from X inactivation in Eutheria: implications for evolution of X chromosome inactivation. Chromosoma 121: 71–78. 10.1007/s00412-011-0343-8 21947602 PMC3260438

[64] Ehrlich M, Lacey M (2013) DNA methylation and differentiation: silencing, upregulation and modulation of gene expression. Epigenomics 5: 553–568. 10.2217/epi.13.43 24059801 PMC3864898

[65] Lund RJ, Narva E, Lahesmaa R (2012) Genetic and epigenetic stability of human pluripotent stem cells. Nat Rev Genet 13: 732–744. 10.1038/nrg3271 22965355

[66] Kucera KS, Reddy TE, Pauli F, Gertz J, Logan JE, et al (2011) Allele-specific distribution of RNA polymerase II on female X chromosomes. Hum Mol Genet 20: 3964–3973. 10.1093/hmg/ddr315 21791549 PMC3177651

[67] Ding Z, Ni Y, Timmer SW, Lee BK, Battenhouse A, et al (2014) Quantitative genetics of CTCF binding reveal local sequence effects and different modes of X-chromosome association. PLoS Genet 10: e1004798 10.1371/journal.pgen.1004798 25411781 PMC4238955

[68] Kung JT, Kesner B, An JY, Ahn JY, Cifuentes-Rojas C, et al (2015) Locus-Specific Targeting to the X Chromosome Revealed by the RNA Interactome of CTCF. Mol Cell 10.1016/j.molcel.2014.12.006 PMC4316200 25578877

[69] Zlatanova J, Caiafa P (2009) CTCF and its protein partners: divide and rule? J Cell Sci 122: 1275–1284. 10.1242/jcs.039990 19386894

[70] Rao SS, Huntley MH, Durand NC, Stamenova EK, Bochkov ID, et al (2014) A 3D Map of the Human Genome at Kilobase Resolution Reveals Principles of Chromatin Looping. Cell 159: 1665–1680. 10.1016/j.cell.2014.11.021 25497547 PMC5635824

[71] Dixon JR, Selvaraj S, Yue F, Kim A, Li Y, et al (2012) Topological domains in mammalian genomes identified by analysis of chromatin interactions. Nature 485: 376–380. 10.1038/nature11082 22495300 PMC3356448

[72] Seitan VC, Faure AJ, Zhan Y, McCord RP, Lajoie BR, et al (2013) Cohesin-based chromatin interactions enable regulated gene expression within preexisting architectural compartments. Genome Res 23: 2066–2077. 10.1101/gr.161620.113 24002784 PMC3847776

[73] Zuin J, Dixon JR, van der Reijden MI, Ye Z, Kolovos P, et al (2014) Cohesin and CTCF differentially affect chromatin architecture and gene expression in human cells. Proc Natl Acad Sci U S A 111: 996–1001. 10.1073/pnas.1317788111 24335803 PMC3903193

[74] Horvath LM, Li N, Carrel L (2013) Deletion of an x-inactivation boundary disrupts adjacent gene silencing. PLoS Genet 9: e1003952 10.1371/journal.pgen.1003952 24278033 PMC3836711

[75] Van Bortle K, Ramos E, Takenaka N, Yang J, Wahi JE, et al (2012) Drosophila CTCF tandemly aligns with other insulator proteins at the borders of H3K27me3 domains. Genome Res 22: 2176–2187. 10.1101/gr.136788.111 22722341 PMC3483547

[76] Dowen JM, Fan ZP, Hnisz D, Ren G, Abraham BJ, et al (2014) Control of cell identity genes occurs in insulated neighborhoods in mammalian chromosomes. Cell 159: 374–387. 10.1016/j.cell.2014.09.030 25303531 PMC4197132

[77] Nora EP, Lajoie BR, Schulz EG, Giorgetti L, Okamoto I, et al (2012) Spatial partitioning of the regulatory landscape of the X-inactivation centre. Nature 485: 381–385. 10.1038/nature11049 22495304 PMC3555144

[78] Li H, Durbin R (2009) Fast and accurate short read alignment with Burrows-Wheeler transform. Bioinformatics 25: 1754–1760. 10.1093/bioinformatics/btp324 19451168 PMC2705234

[79] Langmead B, Trapnell C, Pop M, Salzberg SL (2009) Ultrafast and memory-efficient alignment of short DNA sequences to the human genome. Genome Biol 10: R25 10.1186/gb-2009-10-3-r25 19261174 PMC2690996

