# Supplementary figures and images for: Escape from X Inactivation Varies in Mouse Tissues
Source: PLoS Genet. 2015 Mar 18;11(3):e1005079. doi: 10.1371/journal.pgen.1005079 (PMC4364777; doi:10.1371/journal.pgen.1005079)

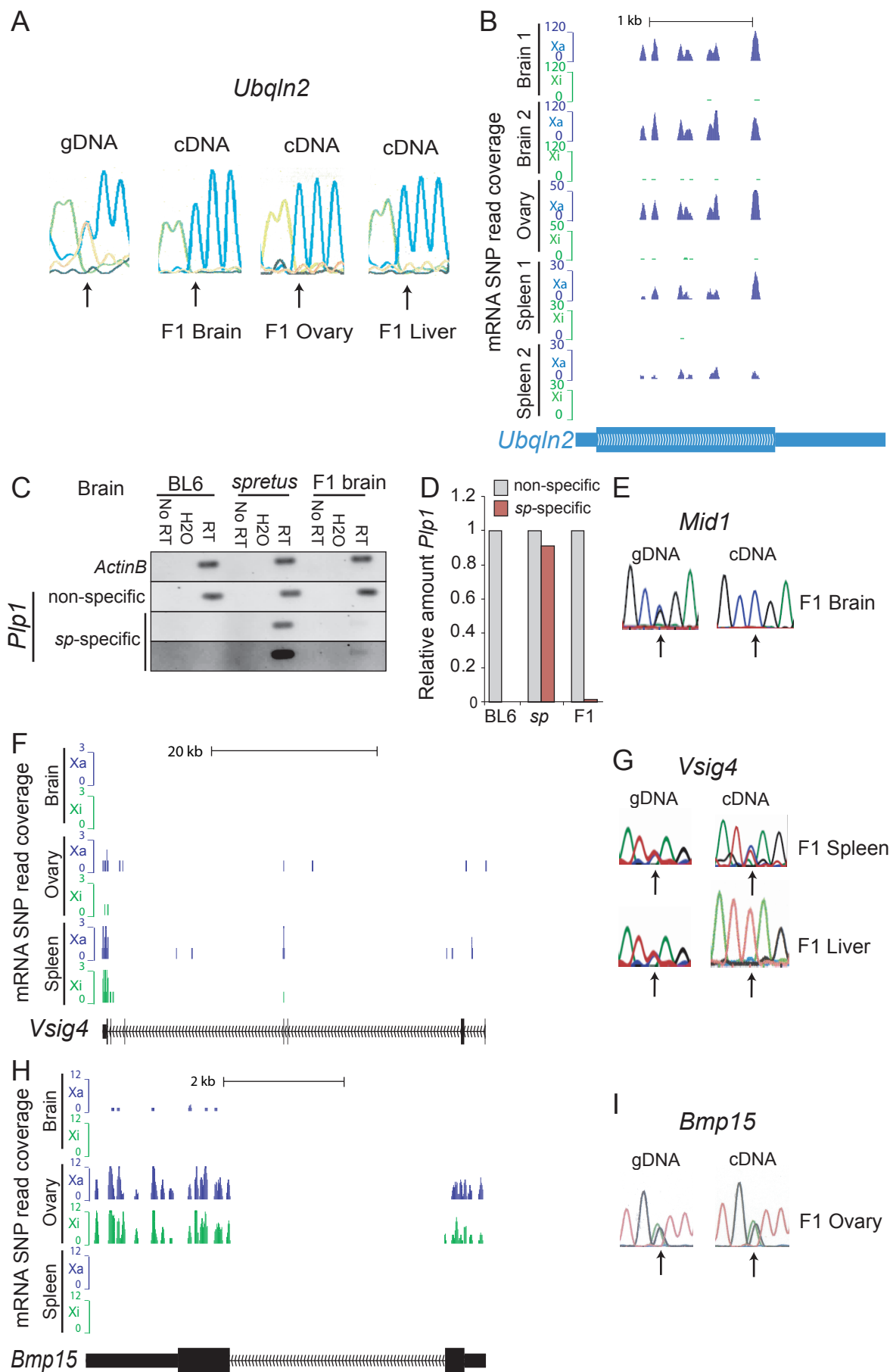

Supplement: S1 Fig — (A) Sanger sequencing of Ubqln2 RT-PCR products confirms XCI skewing in F1 female mice. cDNA tracings show only the BL6 allele in brain, ovary, and spleen, while gDNA tracing confirms heterozygosity at a SNP (C in BL6 and T in spretus). Arrows indicate SNP positions. (B) mRNA SNP read distribution profiles obtained by RNA-seq for Ubqln2 demonstrate the absence of spretus Xi reads in brain, ovary and spleen. Xa SNP reads are in blue and Xi SNP reads in green. (C) Validation of escape from XCI for Plp1 using RT-PCR with species-specific primers. Gel electrophoresis of RT-PCR products using non-species-specific primers and spretus-specific primers (S10 Table) in BL6, spretus, and F1 brain in which the Xi is from spretus. ActinB was used as a control. Control reactions include "No RT" (no reverse transcriptase) and H2O (instead of primers). (D) Xi expression of Plp1 was determined to be 1.5% of total Plp1 expression in F1 brain by gel band quantification measured by Imagej. (E) Mid1 cDNA Sanger sequencing confirms inactivation of the spretus allele in brain, while gDNA tracing shows heterozygosity of Mid1 (C in BL6 and G in spretus). Arrows indicate SNP positions. (F) mRNA SNP read distribution profiles obtained by RNA-seq for Vsig4 a gene that escapes XCI in spleen, but is subject to XCI in liver. Xa SNP reads are in blue and Xi SNP reads in green. (G) Vsig4 cDNA Sanger sequencing tracings confirm bi-allelic expression in spleen but not liver, while gDNA tracings show SNP heterozygosity (T in BL6 and C in spretus). Arrows indicate SNP positions. (H) mRNA SNP read distribution profiles obtained by RNA-seq show bi-allelic expression of Bmp15 in ovary, but not in brain or spleen. Xa SNP reads are in blue and Xi SNP reads in green. (I) Bmp15 cDNA Sanger sequencing tracing confirms escape from XCI for in ovary while gDNA tracing shows SNP heterozygosity (A in BL6 and G in spretus). Arrows indicate SNP positions. (PDF) [file pgen.1005079.s001.pdf]

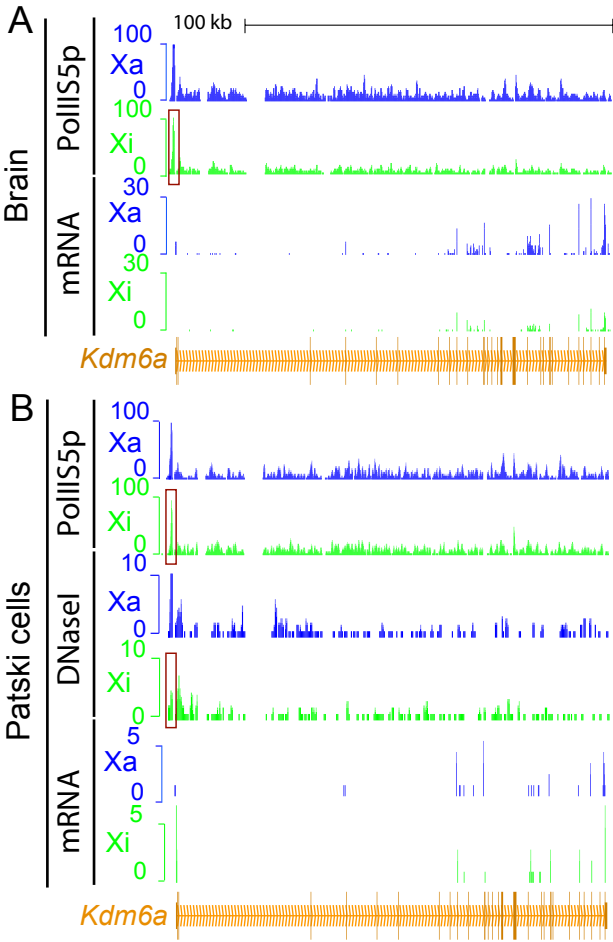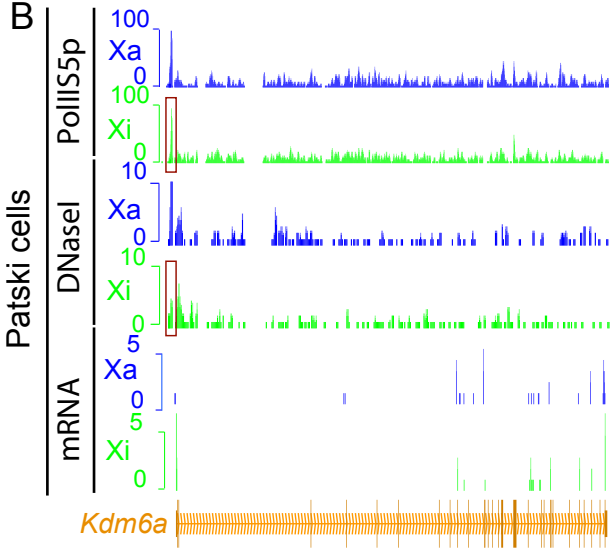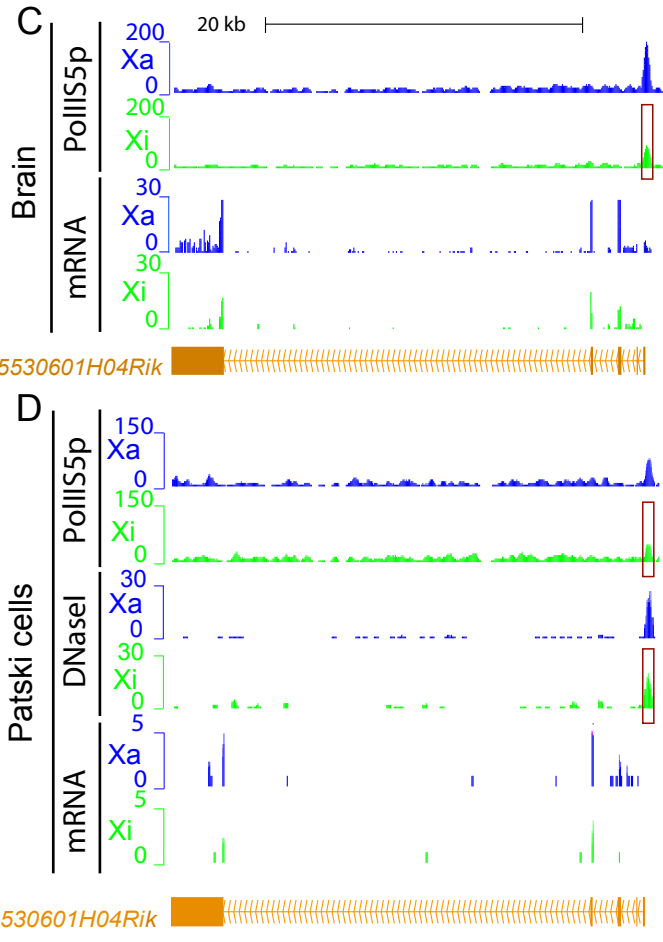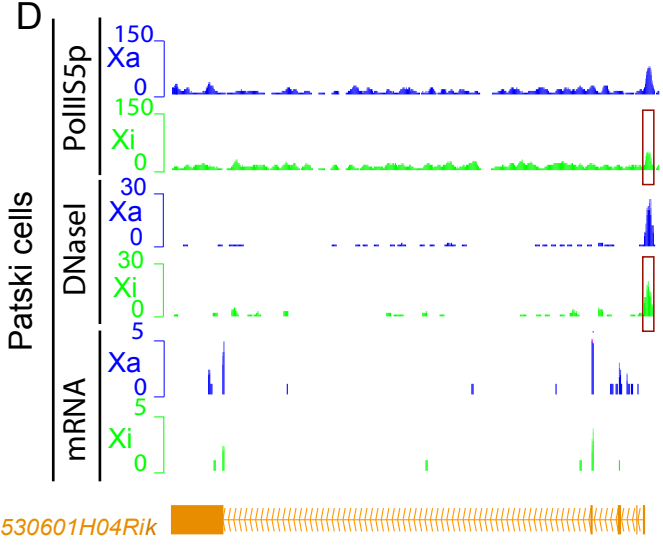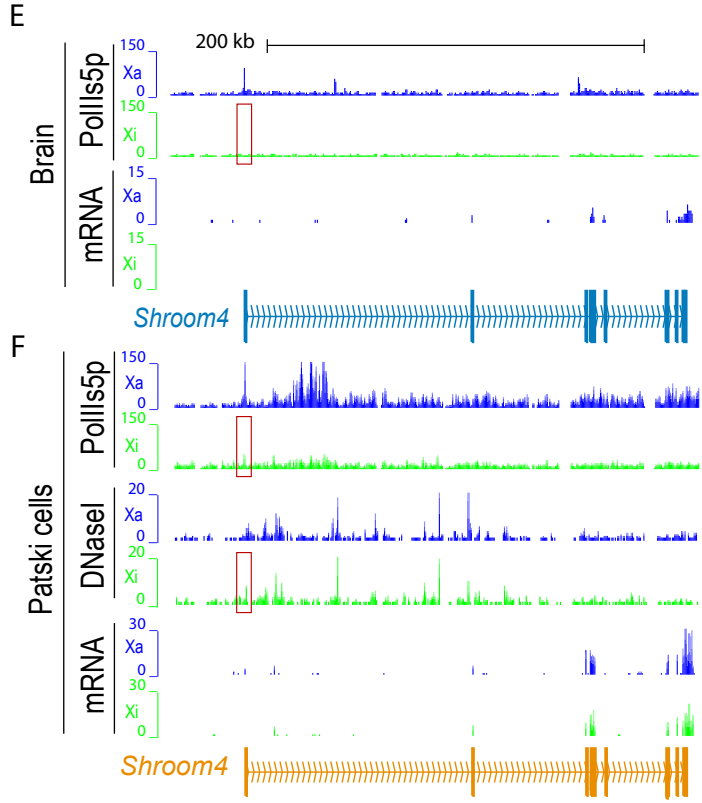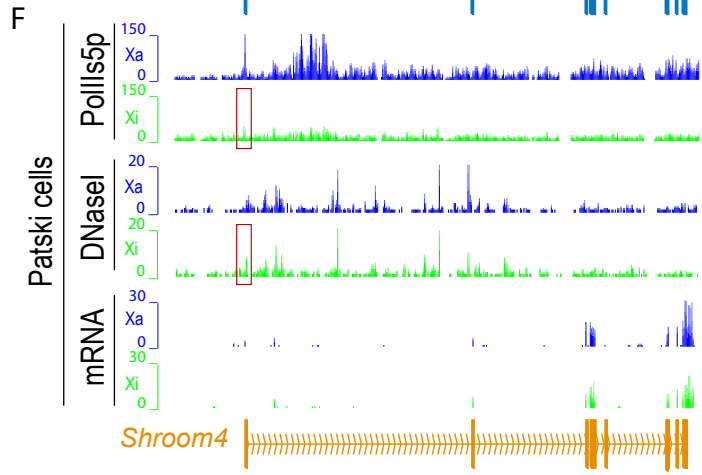

Supplement: S2 Fig — (A, B) Examples of allele-specific PolII-S5p occupancy profiles and expression (mRNA) profiles at Kdm6a, a common escape gene in brain (A) and Patski cells (B). PolII-S5p is enriched at the promoter region (highlighted by a red box) on both the Xa and the Xi. DNase I hypersensitivity tested in Patski cells only is also increased at the promoter region (highlighted by a red box) on both the Xa and Xi. Xa SNP reads are in blue and Xi SNP reads in green. (C, D) Same analysis for the lncRNA 5530601H04Rik, another common escape gene. (E, F) Same analysis for Shroom4, a gene subject to XCI in brain (labeled blue) but that escapes XCI in Patski cells (labeled orange). PolII-S5p is enriched at the promoter region (highlighted by a red box) of Shroom4 on both the Xa and the Xi in Patski cells, whereas enrichment is limited to the Xa in brain. DNase I hypersensitivity tested in Patski cells only is also increased at the promoter region (highlighted by a red box) on both the Xa and Xi. (PDF) [file pgen.1005079.s002.pdf]

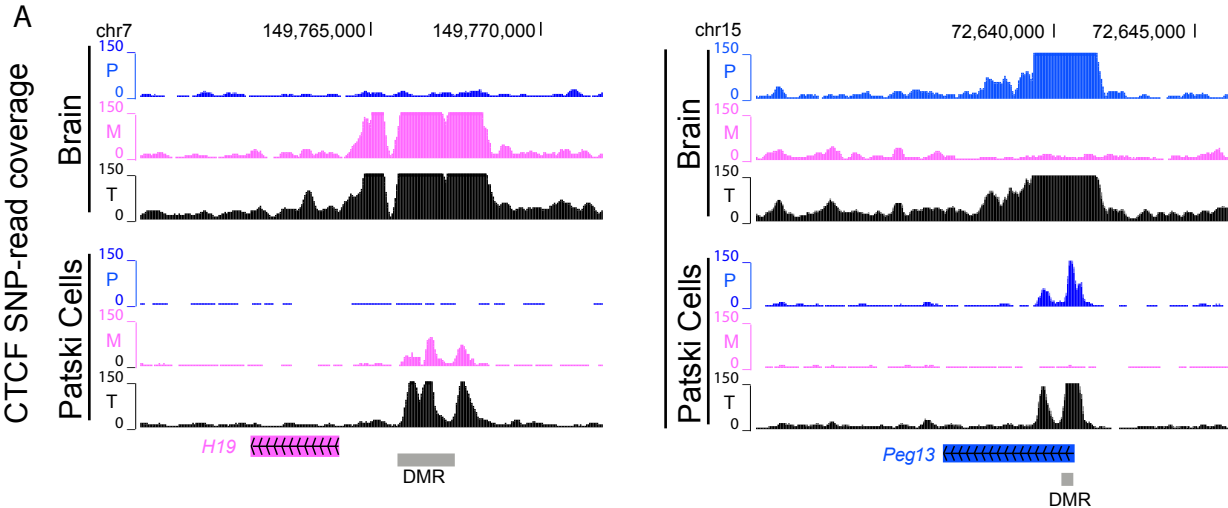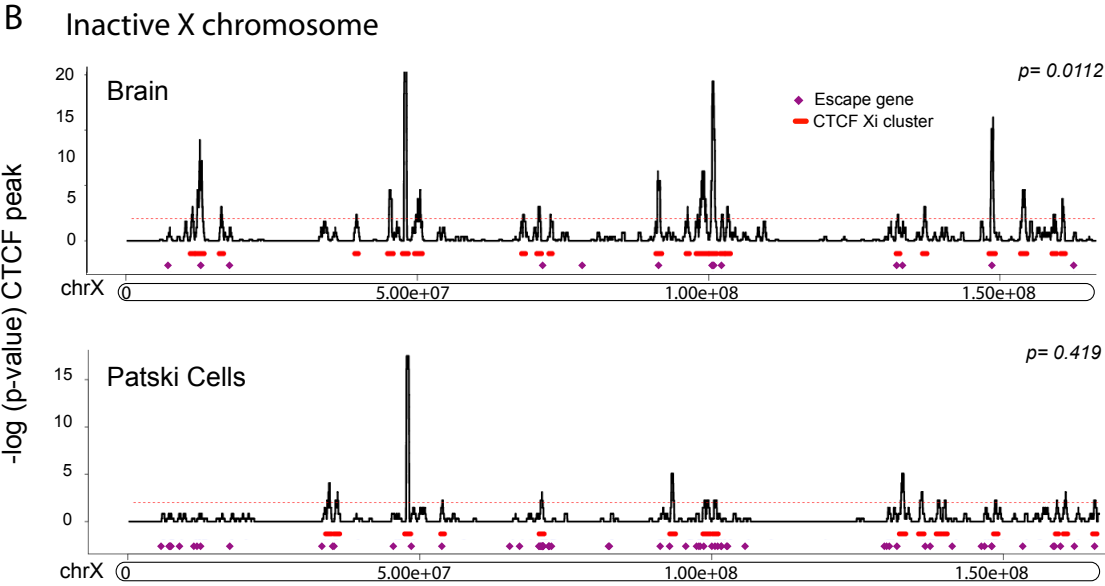

Supplement: S3 Fig — (A) CTCF ChIP-seq analysis in brain and Patski cells at two imprinted regions. On mouse chromosome 7 H19 is only expressed from the maternal allele while Peg13 on mouse chromosome 15 is expressed from the paternal allele. CTCF binding upstream of these genes is high on the allele from which they are expressed, in agreement with a previous study [44]. M, maternal allele and P, paternal allele, T, total reads from both alleles. The differentially methylated regions (DMR) are indicated. (B) Non-promoter significant CTCF Xi-binding clusters were mapped along the Xi in brain and Patski cells (compare to Fig. 6A). After CTCF peaks located around promoters (±1kb from the TSS) were excluded Xi- and both-preferred peaks were determined by a binomial model and used for density analysis. Red bars represent merger of clusters of CTCF Xi-binding peaks, while purple dots represent escape genes. Non-promoter significant Xi-binding CTCF binding clusters tend to co-localize in regions containing escape genes and are more abundant in brain than Patski cells. Horizontal axis represents the Xi in Mb. The vertical axis is the negative log of the calculated binomial p-value [-log (p-value)]. The thin red dashed line represents a 0.01 p-value cutoff. (PDF) [file pgen.1005079.s003.pdf]

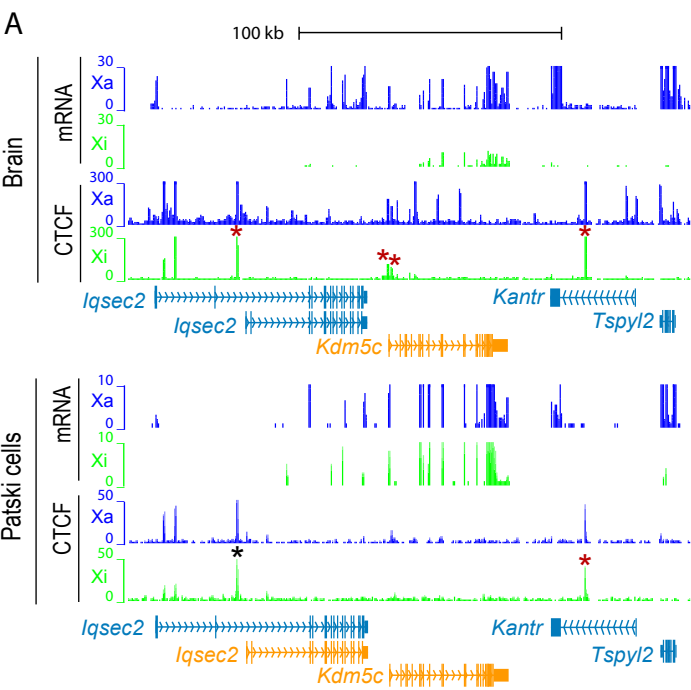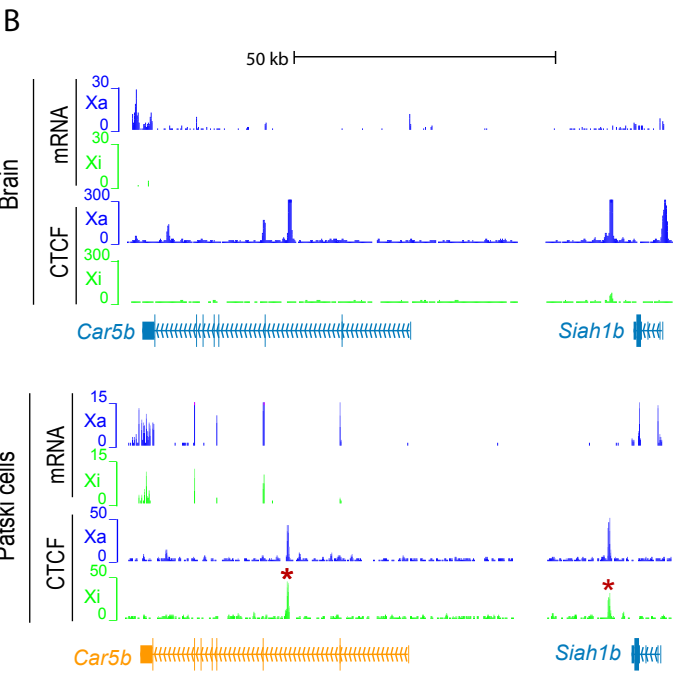

Supplement: S4 Fig — (A) Example of mRNA SNP read distribution profiles and allele-specific CTCF distribution profiles at the Kdm5c-Iqsec2 region in brain and Patski cells (see also Fig. 7). (B) Example of mRNA SNP read distribution profiles and allele-specific CTCF distribution profiles at the Car5b and Siah1b region in brain and Patski cells (see also Fig. 7). RNA-seq read quantification was done by normalizing reads from the Xi to total reads (Xi + Xa) in two biological replicates. Xa SNP reads are in blue and Xi SNP reads in green. Genes that escape XCI are labeled orange and genes subject to XCI blue. (PDF) [file pgen.1005079.s004.pdf]
